# Tumor-derived RAC1^A159V^ mutation promotes an immunosuppressive microenvironment that represses response to immune checkpoint inhibitor

**DOI:** 10.1126/sciadv.aea1212

**Published:** 2025-10-29

**Authors:** Mingjun Cai, Mike Adam, Xin Duan, Fukun Guo, Yi Zheng

**Affiliations:** ^1^ Graduate Program of Development, Stem Cells, and Regenerative Medicine, University of Cincinnati College of Medicine, 3333 Burnet Avenue, Cincinnati, OH 45229, USA.; ^2^ Division of Experimental Hematology and Cancer Biology, Cancer and Blood Diseases Institute, Children’s Hospital Medical Center, and Department of Pediatrics, University of Cincinnati College of Medicine, 3333 Burnet Avenue, Cincinnati, OH 45229, USA.

## Abstract

RAC1^A159V^ is a hotspot mutation associated with poor prognosis in several cancers. By gene editing, we generated endogenous homozygous and heterozygous RAC1^A159V^ mutations, which result in up-regulated RAC1 activity and mammalian target of rapamycin (mTOR) signaling. RAC1^A159V^ tumors grow faster than RAC1^WT^ tumors in immune-proficient mice and are resistant to anti–programmed death protein 1 (PD1). Flow cytometry and scRNA-seq analyses reveal that RAC1^A159V^ cells form “cold” tumors with an immunosuppressive microenvironment and reduced tumor-immune cell interactions. Mechanistically, RAC1^A159V^ up-regulates glycosphingolipid biosynthesis to activate mTORC1 signaling in tumor cells, which in turn increases glycolysis, impairs key chemokine production, and decreases IFNGR1 expression of the tumor cells. mTORC1 inhibition by rapamycin resensitizes the RAC1^A159V^ tumors to anti-PD1 treatment by reversing effects of RAC1^A159V^ mutation. These results demonstrate a mechanism of RAC1^A159V^-driven immune evasion and suggest an approach of combining the targeting of RAC1-mTOR signaling with immune checkpoint inhibitor for the treatment of a type of immune-cold tumors.

## Teaser

The tumor-derived RAC1^A159V^ mutation fosters an immune-cold microenvironment, limiting response to checkpoint immunotherapy.

## INTRODUCTION

Immune checkpoint inhibitors (ICIs) are a transformative class of immunotherapeutic agents that enhance the host immune system’s ability to eliminate cancer cells (*1*). Notably, the ICIs including antibodies against cytotoxic T lymphocyte antigen-4, programmed death protein 1 (PD-1), and programmed death-ligand 1 (PD-L1) have significantly improved clinical outcomes for patients with several cancer types (*2*). However, the overall objective response rate to ICIs remains limited, typically observed in only 10 to 40% of patients of various cancer types (*3*, *4*), highlighting a crucial unmet need in cancer immunotherapy.

CD8^+^ cytotoxic T cells are a major effector cell type in anticancer immune response, and the presence and functionality of CD8^+^ cytotoxic T cells within the tumor immune microenvironment (TIME) are prerequisites for ICI responses (*5*). Current studies have demonstrated a strong correlation between the ICI efficacy and TIME (*6*, *7*). The characteristics of ICI nonresponsive TIME include low immune cell infiltration, high ratio of regulatory T cells (T_reg_ cells) to CD8^+^ cytotoxic T cells, and abnormal tumor-immune cell crosstalk (*7*, *8*). The tumor cell intrinsic effects derived from oncogenic mutations are important regulators of TIME. For example, tumor-driven glycolysis within TIME creates a low-glucose environment, which can impair the effector function of CD8^+^ T cells while promoting T_reg_ cell proliferation, thereby reinforcing an immunosuppressive milieu with reduced ICI effectiveness (*9*, *10*). Dysregulated chemokine production from tumor cells can hinder the infiltration of effector T cells, leading to an “immune-cold” TIME that is poorly responsive to ICIs (*11*, *12*). In addition, aberrant gene expression profiles related to tumor-immune cross-talk, particularly through disrupting interferon-γ (IFN-γ) signaling, can also protect tumor cells from T cell killing and contribute to ICI resistance (*13*).

The Rho guanosine triphosphatase (GTPase) family member RAC1 plays a pivotal role in various critical cellular activities by cycling between inactive guanosine diphosphate–bound and active guanosine 5′-triphosphate (GTP)–bound states (*14*). The hyperactivation of RAC1 is found in various types of tumors (*14*, *15*), and the rational targeting of RAC1 signaling has emerged as a potential anticancer approach (*16*, *17*). RAC1 is involved in cytokine/chemokine secretion, endocytic and exocytic vesicle trafficking, and epithelial-mesenchymal transition, functions closely related to tumor microenvironment remodeling (*18*–*21*). One of the downstream effectors of RAC1, mammalian target of rapamycin (mTOR), promotes tumor cell metabolism including glycolysis and lipid biosynthesis (*22*). This metabolic regulation can change the metabolite composition within TIME and potentially reprogram the metabolism of immune cells (*22*–*24*).

RAC1^A159V^ mutation is a hotspot gain-of-function (GOF) mutation that favors a more stabilized GTP-bound state (*25*, *26*). It is a paralogous to KRAS^A146^ mutation, which most commonly occurs in colon cancer and correlates with poor overall survival (*27*, *28*). Despite of its clinical relevance, studies on RAC1^A159V^ have been largely confined to biochemical analyses (*25*), and its contribution to TIME remains unknown. Here, we investigated the role of RAC1^A159V^ mutation in modulating TIME and tumor response to ICI by using CRISPR-Cas9–generated RAC1^A159V^ tumor cells in mouse models. We found that RAC1^A159V^ creates a “cold” immunosuppressive TIME that renders the tumor highly resistant to anti-PD1. Mechanistically, RAC1^A159V^ activates mTORC1 signaling in tumor cells, leading to enhanced glycolysis, suppressed chemokine production, and reduced expression of IFN-γ receptor 1 that collectively contribute to an immune-evasive tumor phenotype. Treatment with low-dose rapamycin, an mTORC1 inhibitor, sensitized RAC1^A159V^ tumors to anti-PD1 without impairing T cell functions within TIME. Our study provides critical mechanistic insights into the impact of RAC1^A159V^ mutation on TIME and presents a potential approach for overcoming tumor immune evasion in the context of targeted inhibition of aberrant RAC1 signaling.

## RESULTS

### Generation of RAC1^A159V^ mutant MC38 colon cancer cells

**Fig. 1.**
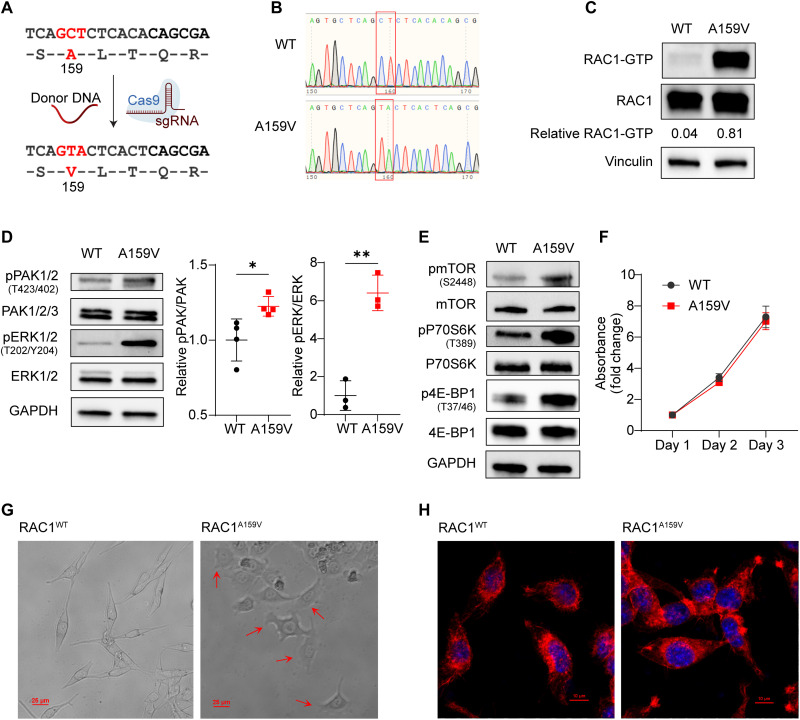
Generation and characterization of RAC1^A159V^ mutant MC38 colon cancer cells. (**A**) Schematic representation of the CRISPR-Cas9–mediated knock-in strategy to generate the RAC1^A159V^ mutation. (**B**) Sanger sequencing of the RAC1 locus in RAC1^WT^ and RAC1^A159V^ cells, confirming the A159V homozygous mutation. (**C**) PAK-PBD pull-down assay to detect RAC1 activity of RAC1^WT^ and RAC1^A159V^ cells. (**D**) Western blot analysis of RAC1 downstream phospho-PAK1/2 (T423/402) and phospho-ERK1/2 (T202/Y204). (**E**) Western blot analysis of mTOR pathway–associated proteins. (**F**) Cell proliferation measured every day by MTS assay. (**G**) Representative bright-field microscopy images illustrating morphology of cells at the leading edge of migration (20×; scale bars, 25 μm). (**H**) Representative immunofluorescence images of F-actin cytoskeleton (60×; scale bar, 10 μm). Data are representative of at least three independent experiments [(C) to (H)]. Data represent means ± SD. Statistical significance determined by unpaired *t* test (D). *P < 0.05 and **P < 0.01. WT, wild type; sgRNA, single guide RNA.

To evaluate the effect of RAC1^A159V^ mutation on tumor cells, we used CRISPR-Cas9–mediated knock-in technology to introduce the A159V mutation to the endogenous RAC1 locus of the MC38 colon cancer cells (Fig. 1A). Sanger sequencing confirmed the successful generation of the desired mutation in the targeted site (Fig. 1B). Three MC38 RAC1^A159V^ cell clones (clones 6, 9, and 14) were obtained after CRISPR-Cas9 gene editing, and they behaved similarly. Data from clone 9 were presented below unless otherwise specified. Unlike previous studies that relied on ectopic overexpression of mutant RAC1 (*29*–*31*), the knock-in mutant allows the investigation of effects by an endogenous RAC1 mutation that better mimics the pathophysiological condition of the tumor cells.

RAC1 activity was assessed by a glutathione S-transferase (GST)-p21-activated kinase 1 (PAK1) pulldown assay, which revealed a notable increase in active GTP-bound RAC1 in RAC1^A159V^ mutant compared to wild type (WT) cells (Fig. 1C). The well-characterized RAC1 signaling effectors, including phospho-PAK1/2 and phospho–extracellular signal–regulated kinase (ERK), were up-regulated in RAC1^A159V^-mutated cells (Fig. 1D). Phospho-mTOR, one of the downstream effectors of RAC1, and the mTOR targets phospho-S6K and phospho–4E-BP1 (p4E-BP1) were up-regulated in A159V mutant cells (Fig. 1E). The elevated mTOR signaling was confirmed in all three MC38 RAC1^A159V^ cell clones (fig. S1). Given the central role of RAC1 in cell proliferation and cytoskeletal dynamics, we examined the impact of the RAC1^A159V^ mutation on cell proliferation and morphology. MC38 RAC1^A159V^ cells did not exhibit significant proliferation advantage in culture (Fig. 1F). However, cells at the leading edge of migration demonstrated notable morphological differences: While WT cells exhibited a characteristic spindle-shaped morphology, mutant cells displayed an extensively spread phenotype characterized by enhanced lamellipodial structures and pronounced membrane ruffling (Fig. 1G, arrows). The phalloidin staining of F-actin showed that dot-shaped focal complexes were formed at the ends of actin cables in RAC1^A159V^ cells, indicating a more active cytoskeleton remodeling (Fig. 1H). These cell morphological features are consistent with increased RAC1-GTP in the cells, further supporting the notion that RAC1^A159V^ is a gain-of-activity mutant.

To assess the generality of these findings, we introduced the RAC1^A159V^ mutation into B16OVA MO4 melanoma cells by the CRISPR-Cas9 approach. Consistent with the observations in MC38 cells, B16OVA RAC1^A159V^ cells exhibited significantly increased RAC1-GTP levels (fig. S2A), up-regulated phospho-PAK1/2 and phospho-ERK (fig. S2B), and up-regulated mTOR activity (fig. S2C). Similar to MC38 cells, B16OVA RAC1^A159V^ cells did not show a proliferation advantage over WT cells in vitro (fig. S2D). Thus, RAC1^A159V^ is a GOF mutation that constitutively activates PAK1/2 and mTOR signaling with significant changes in cell cytoskeleton structures.

### RAC1^A159V^ mutation promotes an immunosuppressive microenvironment and renders tumor cell resistant to anti-PD1

**Fig. 2.**
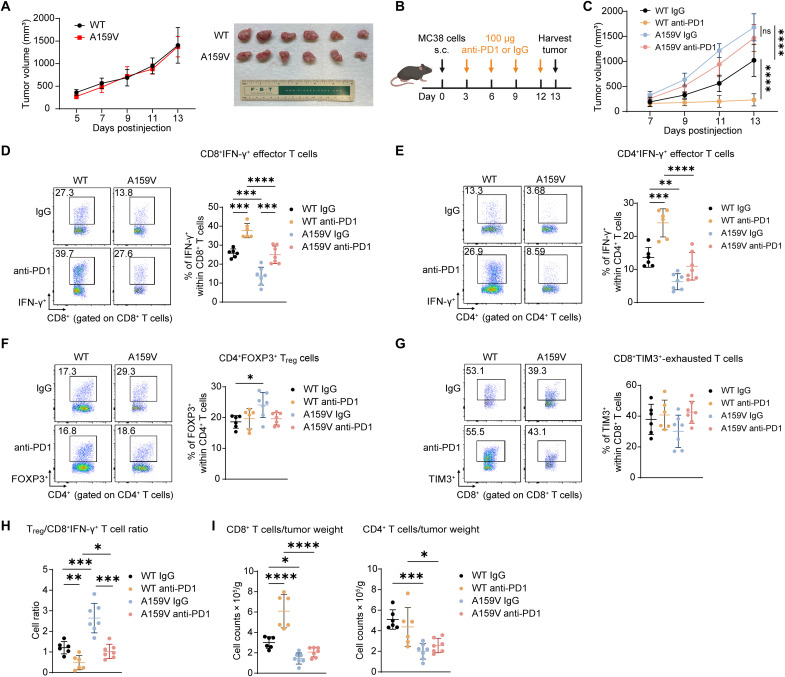
RAC1^A159V^ tumors confer resistance to anti-PD1 treatment with an immunosuppressive TIME. (**A**) MC38 RAC1^WT^ and RAC1^A159V^ cells (2 million) were subcutaneously injected into NSG (NOD-scid IL2Rgamma^null^) mice on day 0. Tumor growth curve (left) and image (right) are shown (*n* = 6 mice per group). (**B**) Schematic illustration of tumor inoculation and anti-PD1 administration. MC38 RAC1^WT^ and RAC1^A159V^ cells (2 million) were subcutaneously injected into C57BL/6 mice on day 0. Anti-PD1 or IgG isotype control (100 μg) was administrated intraperitoneally every 3 days starting from day 3. Tumors were harvested on day 13. (**C**) Tumor growth curve of MC38 RAC1^WT^ and RAC1^A159V^ tumors grown in C57BL/6 mice treated as indicated (*n* = 6 to 7 mice per group). (**D** to **G**) Frequencies of IFN-γ^+^ effector T cells among CD8^+^ T cells (D) and CD4^+^ T cells (E), Treg cells among CD4^+^ T cells (F), and TIM3^+^-exhausted T cells among CD8^+^ T cells (G) within the TIME of tumors in (C). Representative pseudocolor plots (left) and dot plots of cell percentage (right) are shown. (**H**) Ratio of T_reg_ to CD8^+^IFN-γ^+^ T cells within the TIME of tumors in (C). (**I**) Quantification of CD8^+^ T cell (left) and CD4^+^ T cell (right) infiltration as counts per tumor weight within the TIME of tumors in (C). Data are representative of two independent experiments [(B) to (I)]. Data represent means ± SD. Statistical significance determined by two-way ANOVA (C) and one-way ANOVA [(D) to (I)]. *P < 0.05, **P < 0.01, ***P < 0.001, and ****P < 0.0001. s.c., subcutanenous.

To investigate the impact of the tumor-derived RAC1^A159V^ mutation on the TIME, an MC38 syngeneic tumor model was established by subcutaneously injecting WT or RAC1^A159V^ cells to mice. In immunodeficient NSG (NOD-scid IL2Rgamma^null^) mice, tumor growth rates were comparable between WT and RAC1^A159V^ cells (Fig. 2A), consistent with in vitro observations (Fig. 1F). We then assessed tumor growth and response to an ICI in immunocompetent C57BL/6 mice. Mice were randomized into four groups and treated with anti-PD1 or immunoglobulin G (IgG) isotype control every 3 days, commencing on day 3 (Fig. 2B). Unlike the findings in NSG mice, the RAC1^A159V^ tumors exhibited significantly faster growth compared to WT tumors in C57BL/6 mice (Fig. 2C), suggesting that the RAC1^A159V^ mutation contributes to altering interactions with the TIME in addition to modulating tumor cell intrinsic signaling cascades. Furthermore, the RAC1^A159V^ tumors were insensitive to anti-PD1 treatment, whereas WT tumors were highly responsive (Fig. 2C).

Flow cytometry analyses of the tumor-infiltrating lymphocytes (TILs) revealed significant alterations in the immune cell landscape of the RAC1^A159V^ tumors. The gating strategy was shown as fig. S3A. A concomitant decrease in the frequency of CD4^+^IFN-γ^+^ and CD8^+^IFN-γ^+^ effector T cells and an increase in the frequency of CD4^+^FOXP3^+^ T_reg_ cells were observed in RAC1^A159V^ tumors compared to WT tumors (Fig. 2, D to F). Notably, the frequency of CD8^+^TIM3^+^-exhausted T cells remained unchanged (Fig. 2G). The elevated T_reg_/CD8^+^IFN-γ^+^ effector T cell ratio within the RAC1^A159V^ tumors indicated a more immunosuppressive microenvironment (Fig. 2H). Anti-PD1 treatment effectively increased the frequency of CD4^+^IFN-γ^+^ effector T cells in WT tumors but had a limited effect in RAC1^A159V^ tumors (Fig. 2E). Although anti-PD1 treatment enhanced the frequency of IFN-γ^+^ effector T cells within the CD8^+^ T cell population in both WT and RAC1^A159V^ tumors, the effect on the frequency of CD8^+^IFN-γ^+^ effector T cells within the total CD45^+^ immune cell population was more pronounced in WT tumors (Fig. 2D and fig. S3, B and C). This is likely due to the different impact of anti-PD1 on CD4^+^ and CD8^+^ T cells: Specifically, while anti-PD1 did not significantly alter the frequency of CD4^+^ T cells, it led to a significant increase in the frequency of CD8^+^ T cells only in WT tumors, but not in RAC1^A159V^ tumors (fig. S3, D and E). A significant reduction in both CD8^+^ and CD4^+^ T cell infiltration was apparent in RAC1^A159V^ tumors, further suggesting a shift toward a cold tumor caused by the RAC1^A159V^ mutation that potentially contributes to the diminished responsiveness to anti-PD1 treatment (Fig. 2I).

Given the critical role of myeloid cells in TIME modulation (*32*, *33*), their infiltration within MC38 WT and RAC1^A159V^ tumors was also analyzed. Similar to the observations for T cells, the infiltration of CD11b^+^ myeloid cells, including CD11b^+^F4/80^+^ macrophages and CD11b^+^Ly-6G/Ly-6C^+^ neutrophils, were decreased in RAC1^A159V^ tumors (fig. S4, A to C). Also, M2/M1 macrophage ratio increased in RAC1^A159V^ tumors (fig. S4D), supporting an immunosuppressive TIME.

In addition to the MC38 colon cancer model, an orthotopic B16OVA melanoma model was also generated by intradermally injecting B16OVA RAC1^WT^ and RAC1^A159V^ homozygous cells (*34*, *35*), and the tumors were treated with IgG or anti-PD1. Consistent with observations in the MC38 model, B16OVA RAC1^A159V^ tumors exhibited resistance to anti-PD1 (fig. S5, A and B).

Together, these data indicate that tumor-derived RAC1^A159V^ mutation renders tumor unresponsive to anti-PD1 treatment, and this is likely due to the decreased immune cell infiltration and an immunosuppressive TIME caused by RAC1^A159V^ mutation.

### scRNA-seq reveals that RAC1^A159V^ produces a cold tumor with reduced cell-cell interactions in TIME

**Fig. 3.**
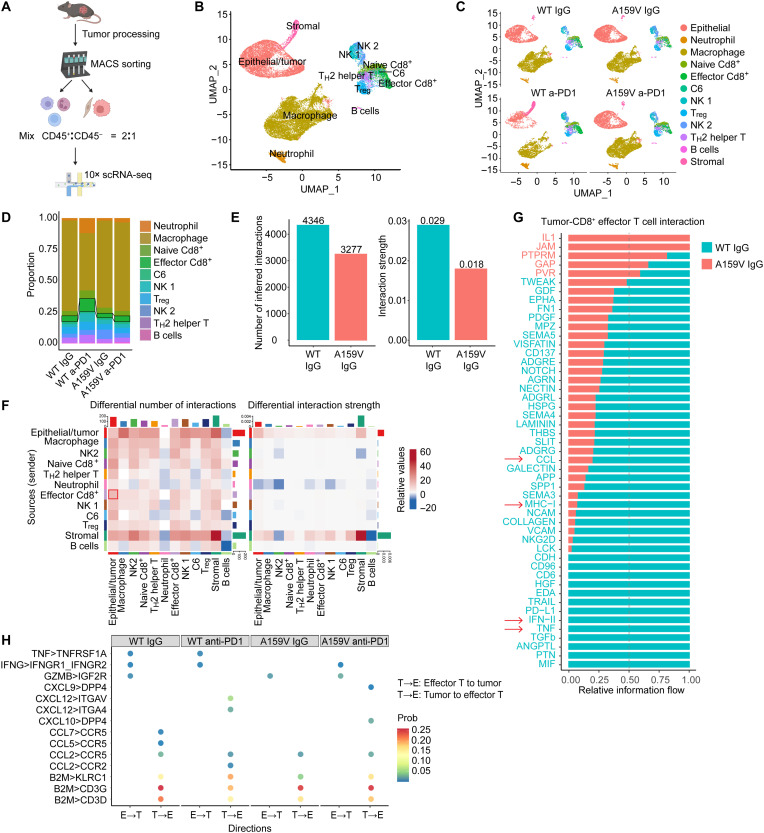
scRNA-seq reveals that RAC1^A159V^ tumors have reduced infiltration of CD8^+^ effector T cells and impaired cellular interactions within the TIME. (**A**) Schematic representation of the sample preparation workflow for scRNA-seq. (**B**) Uniform Manifold Approximation and Projection (UMAP) clustering of all cells from tumor tissues, identifying 12 distinct cell clusters, with each cluster represented by a unique color. (**C**) UMAP of tumor and immune cells from RAC1^WT^ and RAC1^A159V^ tumor tissues with IgG or anti-PD1 treatment. (**D**) Bar plot showing relative differences in immune cell infiltration in RAC1^WT^ and RAC1^A159V^ tumors. (**E** and **F**) Number of interactions (left) and interaction strength (right) among all cell clusters in WT IgG and A159V IgG samples, represented by bar plots (E) and heatmaps (F). (**G**) Relative information flow of significant interactions between tumor cells and CD8^+^ effector T cells in WT IgG and A159V IgG samples. (**H**) Selected ligand-receptor interactions between tumor cells and CD8^+^ effector T cells.

To further understand the changes in TIME caused by the RAC1^A159V^ mutation, next we performed single-cell RNA sequencing (scRNA-seq) on MC38 RAC1^WT^ and RAC1^A159V^ tumors that were treated with anti-PD1 or IgG isotype control (Fig. 3A). We identified several major cell clusters, including T cells, B cells, macrophages, neutrophils, natural killer (NK) cells, and MC38 epithelial tumor cells (epithelial/tumor) based on established cell-type marker annotations (Fig. 3B and fig. S6A). Consistent with the flow cytometry findings, scRNA-seq analyses revealed a diminished abundance of CD8^+^ effector T cells within the RAC1^A159V^ IgG tumor compared to WT IgG tumor, while anti-PD1 significantly augmented CD8^+^ effector T cell populations in WT tumors but exerted a limited effect in RAC1^A159V^ tumors (Fig. 3, C and D). Similarly, IFN-γ^+^, tumor necrosis factor–α positive (TNF-α^+^), and CD69^+^ T cells were reduced in RAC1^A159V^ IgG tumors and did not exhibit further increase after anti-PD1 treatment (fig. S6, B to D).

To determine whether distinct cell-cell interaction patterns within the TIME may contribute to the immunosuppressive TIME- and ICI-resistant phenotype, we conducted intercellular communication analyses on the scRNA-seq data to explore possible changes in cell-cell interactions induced by RAC1^A159V^ mutation. We compared the A159V IgG tumor with WT IgG tumor and observed that both the predicted number of receptor-ligand interactions and the predicted differential interaction strength were significantly lower in RAC1^A159V^ tumor compared to WT tumor (Fig. 3E). Specifically, when the alterations in individual cellular interaction pairs were visualized using circos plots and heatmaps, where red denotes up-regulation and blue represents down-regulation (WT IgG versus A159V IgG), epithelial tumor cells in the WT samples exhibited a greater number and intensity of interactions than epithelial tumor cells in the RAC1^A159V^ samples (Fig. 3F and fig. S7A); the number of signaling secreted from CD8^+^ effector T cells, the most potent tumor-killing immune cells, to epithelial tumor cells was more abundant in WT tumors (Fig. 3F). Furthermore, the number of interactions between tumor cells and each immune cell type (except B cells) was diminished in RAC1^A159V^ tumors, regardless of communication directions (fig. S7B). These findings suggest that RAC1^A159V^ mutation impairs the ability of tumor cells to engage in effective interactions with immune cells, potentially shielding tumor cells from immune cell engagement.

To further dissect specific cell-cell interaction pathways, we performed a relative information flow analysis across all cell type pairs based on a summation of probability of pathway communication. The results revealed a significant decrease in multiple pathways within RAC1^A159V^ tumors (fig. S7C). Notably, CCL and CXCL interactions, which are crucial for immune cell migration, and IFN and TNF signaling, which play critical roles in tumor killing, were markedly diminished in RAC1^A159V^ tumors compared to WT tumors (fig. S7C). To verify a possible reduction of T cell chemotaxis in RAC1^A159V^ tumors, we first assessed the expression of key chemokines in tumor cells. There are fewer tumor cells in RAC1^A159V^ IgG group that express various chemokines compared with those in WT IgG group (fig. S7D), and the percent of chemokine expressing tumor cells increased by anti-PD1 in WT tumors but not in RAC1^A159V^ tumors (fig. S7D). Similarly, chemotaxis tumor cell-immune cell interaction pairs were inhibited in RAC1^A159V^ IgG group and were unresponsive to anti-PD1 in RAC1^A159V^ tumors compared to WT group (fig. S7E). Subsequent analyses on the interactions between tumor cells and CD8^+^ effector T cells saw that the CCL chemotaxis signaling, major histocompatibility complex-I antigen presentation signaling, and IFN-II/TNF tumor killing signaling were all reduced in RAC1^A159V^ tumors (Fig. 3G). A detailed ligand-receptor pair analysis further confirmed these findings (Fig. 3H). These single-cell analyses show that the communication between tumor cells and immune cells was significantly decreased and unresponsive to anti-PD1 in RAC1^A159V^ tumor, demonstrating that RAC1^A159V^ mutation promotes a “cold tumor” with an immunosuppressive microenvironment.

### Bulk RNA-seq identifies altered metabolism, chemotaxis, and IFN signaling in RAC1^A159V^ tumor cells

**Fig. 4.**
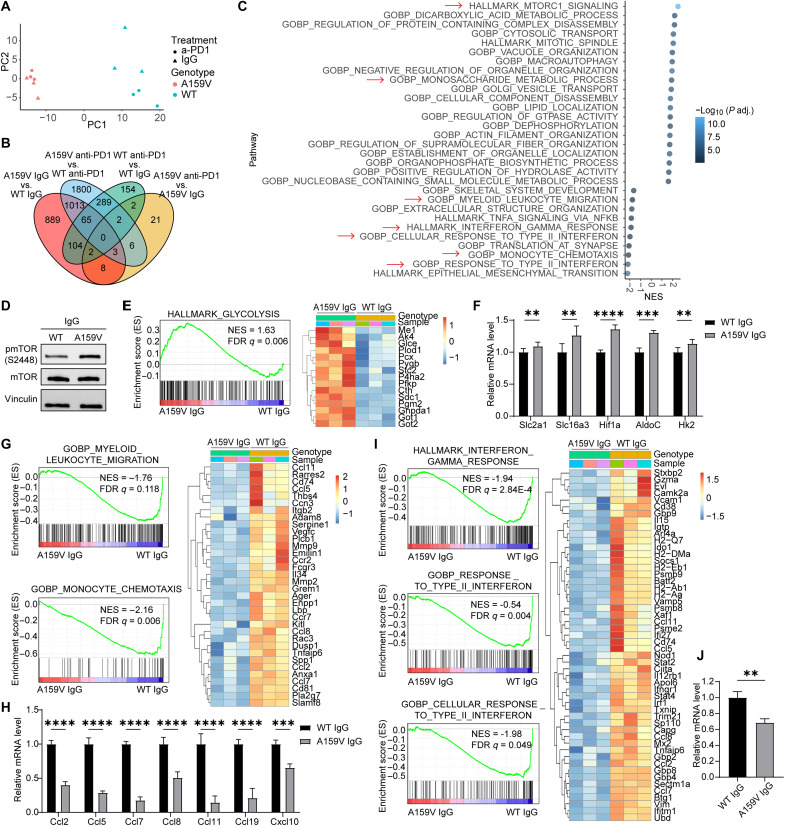
Bulk RNA-sequencing reveals altered signaling pathways in RAC1^A159V^ tumor cells. (**A**) PCA plot of tumor cells isolated from MC38 RAC1^WT^ and RAC1^A159V^ tumor tissues treated with IgG or anti-PD1 (*n* = 3 mice per group). (**B**) Veen diagram of differentially expressed genes (DEGs) in tumor cells (log 2 fold change > 0.3). (**C**) GSEA of the top 20 up-regulated and 10 down-regulated pathways in A159V IgG tumor cells versus WT IgG tumor cells, ranked by normalized enrichment score (NES). (**D**) Representative Western blot analysis of phospho-mTOR in tumor cells isolated from WT IgG and A159V IgG tumor tissues. (**E**) GSEA enrichment plot for gene set HALLMARK_GLYCOLYSIS (left) and heatmap of significant enrichment genes (right) in tumor cells isolated from WT IgG and A159V IgG tumor tissues. (**F**) RT-qPCR analysis of glycolysis-related gene expression in tumor cells isolated from WT IgG and A159V IgG tumor tissues (*n* = 3). (**G**) GSEA enrichment plot for gene set GOBP_MYELOID_LEUKOCYTE_MIGRATION and GOBP_MONOCYTE_CHEMOTAXIS (left) and heatmap of significant enrichment genes (right) in tumor cells isolated from WT IgG and A159V IgG tumor tissues. (**H**) RT-qPCR analysis of selected chemokine gene expression in tumor cells isolated from WT IgG and A159V IgG tumor tissues (*n* = 3). (**I**) GSEA enrichment plot for gene set HALLMARK_INTERFERON_GAMMA_RESPONSE, GOBP_RESPONSE_TO_TYPE_II_INTERFERON, and GOBP_CELLULAR_RESPONSE_TO_TYPE_II_INTERFERON (left), and heatmap of significant enrichment genes (right) in tumor cells isolated from WT IgG and A159V IgG tumor tissues. (**J**) RT-qPCR analysis of Ifngr1 gene expression in tumor cells isolated from WT IgG and A159V IgG tumor tissues (*n* = 3). Data represent means ± SD. Statistical significance determined by two-way ANOVA [(F) and (H)] and unpaired *t* test (J). **P < 0.01, ***P < 0.001, and ****P < 0.0001.

To comprehensively characterize the tumor intrinsic molecular alterations by RAC1^A159V^ leading to TIME changes, we performed deeper RNA-seq analysis on tumor cells isolated from in vivo tumor samples. Principal components analysis (PCA) and hierarchical clustering of the RNA-seq data demonstrated robust reproducibility between biological replicates, indicating high data quality (Fig. 4A). Notably, the PCA plot revealed distinct clustering of RAC1^WT^ IgG and RAC1^WT^ anti-PD1 groups, whereas the RAC1^A159V^ anti-PD1 group clustered closely with the RAC1^A159V^ IgG group, suggesting that anti-PD1 exerts limited effect on the transcriptional landscape of RAC1^A159V^ tumors (Fig. 4A). This was further validated by a differentially expressed gene (DEG) analysis. RAC1^A159V^ mutation significantly altered the expression of 2084 genes compared to WT tumors (A159V IgG versus WT IgG). Anti-PD1 treatment significantly modulated the expression of 618 genes in WT tumors (WT a-PD1 versus WT IgG), while it only affected 44 genes in RAC1^A159V^ tumors (A159V a-PD1 versus A159V IgG) (Fig. 4B).

Among the top 25 up-regulated and down-regulated genes in RAC1^A159V^ IgG-treated tumors compared with WT IgG tumors (fig. S8), a set of metabolic genes—including Slc5a4a, a glucose sensor, and Prg4, a gene associated with glucose utilization (*36*, *37*)—were up-regulated in RAC1^A159V^ tumors, while a set of genes involved in immune functions such as Ifitm1, Pla2g7, S100a4, Gbp4, and H2-Aa, which are crucial for establishing a “hot” tumor microenvironment (*38*–*44*), were down-regulated.

For a deeper insight into the functional consequences of the transcriptional changes, we performed Gene Set Enrichment Analysis (GSEA) on the DEGs. The “HALLMARK_MTORC1_SIGNALING” pathway appeared as the most significantly enriched pathway in A159V tumors. Consistent with the RNA-seq data, Western blotting confirmed the elevated mTOR signaling in RAC1^A159V^ tumors in both in vitro culture and in vivo tumor experimental settings (Figs. 1E and 4D). In addition, “GOBP_MONOSACCHARIDE_METABOLIC_PROCESS” was among the top up-regulated gene sets (Fig. 4C). Specifically, the “HALLMARK_GLYCOLYSIS” gene set was enriched, highlighting an increase in glycolytic activity of the RAC1^A159V^ tumor cells (Fig. 4E). Reverse transcription quantitative polymerase chain reaction (RT-qPCR) analysis confirmed up-regulation of key glycolytic genes, including glucose transporter 1 (Glut1/Slc2a1), monocarboxylate transporter 4 (Mct4/Slc16a3), Hif1α, Aldolase C (AldoC), and Hexokinase 2 (Hk2) (Fig. 4F and fig. S9A). Gene sets related to immune cell recruitment—such as “GOBP_MYELOID_LEUKOCYTE_MIGRATION” and “GOBP_MONOCYTE_CHEMOTAXIS”—were down-regulated, suggesting impaired chemotactic processes in A159V tumors (Fig. 4, C and G). In line with this, the expression of chemokines including Ccl2, Ccl5, Ccl7, Ccl8, Ccl11, Ccl19, and Cxcl10 were significantly decreased in RAC1^A159V^ tumors (Fig. 4H and fig. S9B). Gene sets associated with IFN-γ response—including “HALLMARK_INTERFERON_GAMMA_RESPONSE,” “GOBP_RESPONSE_TO_TYPE_II_INTERFERON,” and “GOBP_CELLULAR_RESPONSE_TO_TYPE_II_INTERFERON”—were also down-regulated in RAC1^A159V^ tumor cells (Fig. 4, C and I), suggesting that the RAC1^A159V^ mutation protects tumor cells from IFN-γ–mediated tumor killing. Given that IFN-γ responses are receptor-mediated signaling through IFN-γ receptor (*45*), we further investigated whether IFN-γ receptor is defective in RAC1^A159V^-mutated tumor cells. RT-qPCR revealed decreased IFN-γ receptor 1 (IFNGR1) expression in both in vitro and in vivo RAC1^A159V^ tumor cells (Fig. 4J and fig. S9C). Flow cytometry further confirmed the decreased IFNGR1 cell surface level in RAC1^A159V^ tumor cells (fig. S9D). The top 25 up-regulated and down-regulated genes following anti-PD1 treatment in WT tumors exhibited no significant change upon anti-PD1 treatment in RAC1^A159V^ tumors (fig. S10A). Notably, the expression of genes involved in glycolysis, chemotaxis, and response to IFN-γ were altered by anti-PD1 in WT tumor cells but remained unchanged in RAC1^A159V^ tumor cells (fig. S10, B to D). These findings further support the notion that anti-PD1 treatment lacks efficacy in tumors harboring the A159V mutation.

Overall, these RNA-seq analyses revealed that the RAC1^A159V^ mutation profoundly alters the cellular metabolic program specifically in enhancing glycolysis and suppresses chemokine transcription and response to IFN-γ.

### RAC1^A159V^ activates tumor cell glycolysis, impairs chemokine production, and inhibits IFNGR1 expression via mTORC1

**Fig. 5.**
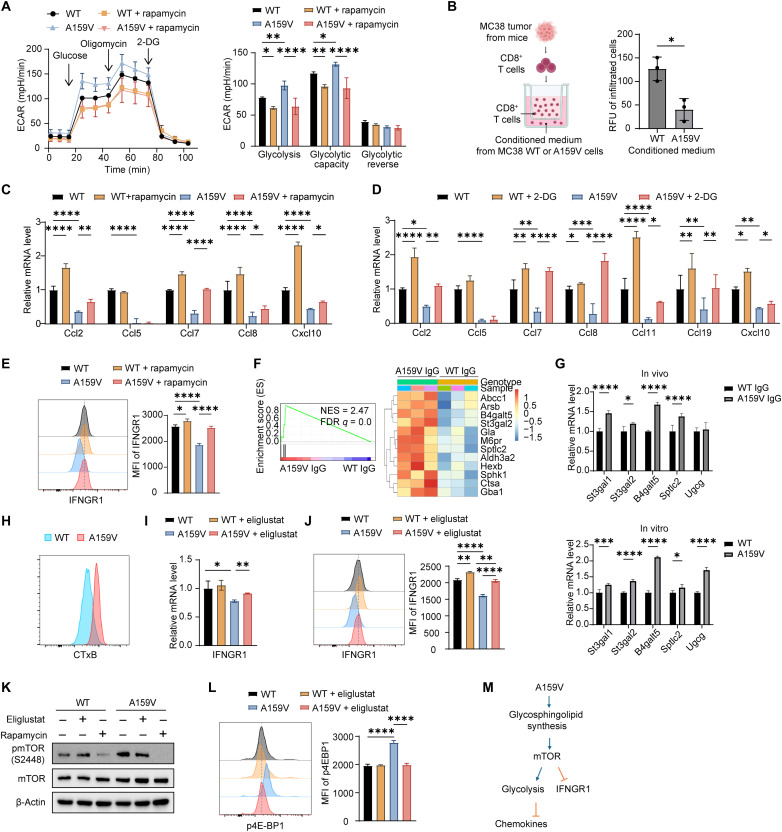
A RAC1^A159V^-sphingolipid-mTORC1 signaling axis promotes glycolysis, suppresses chemokine production, and down-regulates IFNGR1 expression in tumor cells. (**A**) Seahorse glycolysis stress test in RAC1^WT^ and RAC1^A159V^ cells treated with or without rapamycin (*n* = 3). (**B**) Chemotaxis assay of CD8^+^ T cells toward conditioned medium from RAC1^WT^ and RAC1^A159V^ cells. Schematic diagram (left) and quantification of migrated cells (right) are shown (*n* = 3). (**C** and **D**) RT-qPCR analysis of chemokines in RAC1^WT^ and RAC1^A159V^ cells treated with or without rapamycin (C) and 2-DG (D) (*n* = 3). (**E**) Flow cytometry analysis of cell surface IFNGR1 expression in RAC1^WT^ and RAC1^A159V^ cells treated with or without rapamycin. Representative histogram (left) and MFI summary (right) are shown (*n* = 3). (**F**) GSEA plot and heatmap of significant enrichment genes for REACTOME_SPHINGOLIPID_METABOLISM in tumor cells isolated from WT IgG and A159V IgG tumor tissues. (**G**) RT-qPCR analysis of sphingolipid biosynthetic enzyme genes in tumor cells isolated from WT IgG and A159V IgG tumor tissues (top) and cultured WT and A159V tumor cells (bottom) (*n* = 3). (**H**) Flow analysis of cell membrane glycosphingolipid levels in RAC1^WT^ and RAC1^A159V^ cells using CTxB. (**I** and **J**) RT-qPCR analysis of Ifngr1 gene expression (I) and flow cytometry analysis of cell surface IFNGR1 expression (J) in RAC1^WT^ and RAC1^A159V^ cells treated with or without eliglustat (*n* = 3). MFI, mean fluorescence intensity. (**K**) Western blot analysis of phospho-mTOR in RAC1^WT^ and RAC1^A159V^ cells untreated and treated with rapamycin or eliglustat. (**L**) Flow cytometry analysis of p4E-BP1 in RAC1^WT^ and RAC1^A159V^ cells treated with or without eliglustat. Representative histogram (left) and MFI summary (right) are shown (*n* = 3). (**M**) Schematic of the signaling pathway induced by RAC1^A159V^ mutation in tumor cells. Data represent means ± SD. Statistical significance determined by two-way ANOVA [(A), (C), (D), and (G)], unpaired *t* test (B), and one-way ANOVA [(E), (I), (J), and (L)]. *P < 0.05, **P < 0.01, ***P < 0.001, and ****P < 0.0001.

To assess the functional consequences of the RAC1^A159V^ mutation as predicted by the above RNA-seq analyses, we performed glycolytic stress tests on WT and RAC1^A159V^ cells. The RAC1^A159V^ cells showed enhanced glycolysis and increased glycolysis capacity under conditions of mitochondrial adenosine 5′-triphosphate (ATP) inhibition in RAC1^A159V^ cells (Fig. 5A). To investigate the impact of RAC1^A159V^ on immune cell chemotaxis, we isolated CD8^+^ T cells, a critical effector population in antitumor immunity, from MC38 tumors, and performed a chemotaxis assay using conditioned medium collected from MC38 WT or RAC1^A159V^ cells after 2 days of culture. Notably, using isolated CD8^+^ T cells that contain ~20% CD69^+^-activated T cells, we observed a significant reduction in the T cell migration toward conditioned medium derived from RAC1^A159V^ cells versus WT cells, suggesting that RAC1^A159V^-regulated chemokines affect T cell infiltration within the tumor microenvironment (Fig. 5B).

Given the robust activation of mTORC1 signaling by the RAC1^A159V^ mutation, we hypothesized that the metabolic and immunomodulatory alterations seen in gene expression were mediated by elevated mTORC1 activity. Treatment with rapamycin, an mTORC1 inhibitor, effectively suppressed glycolytic activity in both WT and RAC1^A159V^ cells (Fig. 5A) while concomitantly increasing chemokine production (Fig. 5C). To further explore the interplay between up-regulated glycolysis and reduced chemokine expression, we treated cells with 2-deoxyglucose (2-DG), a glucose analog that inhibits glycolysis. 2-DG treatment also resulted in increased chemokine production, similar as observed with rapamycin (Fig. 5D), suggesting that mTORC1 signaling suppresses chemokine expression predominantly through its effects on glycolysis. Furthermore, rapamycin treatment significantly increased IFNGR1 cell surface expression in RAC1^A159V^ cells, restoring it to the level comparable to that in WT cells. However, this effect of rapamycin appeared minimal in WT cells (Fig. 5E).

Given that glycosphingolipid biosynthesis reduces surface level of IFNGR1 and facilitates immune evasion in Kirsten rat sarcoma virus (KRAS)-driven cancer (*46*), we hypothesized that an analogous mechanism involving glycosphingolipid metabolism contributes to the suppression of IFNGR1 in the context of the RAC1^A159V^ mutation. Supporting this hypothesis, the RNA-seq of tumor cells suggested an alteration of sphingolipid metabolism pathway in RAC1^A159V^ tumor cells compared with WT tumor cells (Fig. 5F). RT-qPCR of both in vitro and in vivo tumor cells also revealed significant up-regulation of key enzymes involved in glycosphingolipid biosynthesis, including St3gal1, St3gal2, B4galt5, Sptlc2, and Ugcg, in A159V-mutated cells (Fig. 5G). Flow cytometry analyses showed that the plasma membrane glycosphingolipid level was up-regulated in RAC1^A159V^ cells using choleratoxin B (CTxB) staining (Fig. 5H). To validate the importance of glycosphingolipid biosynthesis for IFNGR1 expression, eligustat, a small-molecule inhibitor of UGCG, was used to deplete glycosphingolipids and their precursors (*46*, *47*). The transcriptional and surface expression of IFNGR1 was restored in A159V cells after an eligustat treatment (Fig. 5, I and J), supporting the involvement of glycosphingolipid biosynthesis in IFNGR1 regulation. Given that different glycosphingolipids and sphingolipids—including glucosylceramide, ceramide 1-phosphate, and ceramide—have the ability to modulate mTOR signaling (*48*–*51*), we next examined whether mTOR activation in RAC1^A159V^ tumor cells is mediated by the observed up-regulation of glycosphingolipid biosynthesis. The pharmacological inhibition of glycosphingolipid biosynthesis using eliglustat resulted in the down-regulation of p-mTOR and its downstream effector p-4E-BP1, in RAC1^A159V^ mutant tumor cells but not in WT cells (Fig. 5, K and L). These findings implicate a unique regulatory role of glycosphingolipids in mTOR signaling in the context of the RAC1^A159V^ mutation.

The above findings were further corroborated by the analyses of two additional MC38 RAC1^A159V^ clones (fig. S11) and the B16OVA RAC1^A159V^ cells (fig. S12), which consistently demonstrate a dysregulation of tumor cell glycolysis, chemotaxis, and IFNGR1 expression by the RAC1^A159V^ mutation. Together, these findings provide strong evidence for the tumor-intrinsic molecular alterations induced by the RAC1^A159V^ mutation that promote tumor cell glycolysis, suppress chemokine production, and reduce cell surface IFNGR1 via glycosphingolipid mediated mTOR activation (Fig. 5M). They may collectively contribute to an immunosuppressive TIME that results in compromised immunotherapy efficacy.

### Rapamycin, a mTORC1 inhibitor, sensitizes RAC1^A159V^ tumor to anti-PD1 treatment

**Fig. 6.**
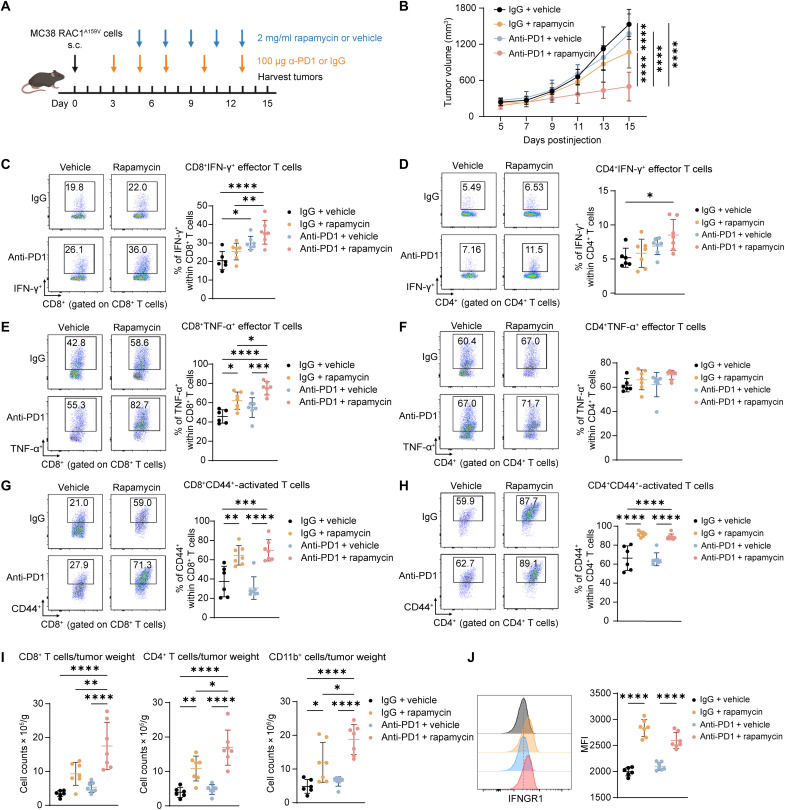
Inhibition of mTORC1 by a low-dose rapamycin sensitizes RAC1^A159V^ tumors to anti-PD1 treatment. (**A**) Schematic illustration of tumor inoculation and drug administration. MC38 RAC1^A159V^ cells (2 million) were subcutaneously injected into C57BL/6 mice on day 0. Anti-PD1 or IgG isotype control (100 μg) was administrated intraperitoneally every 3 days starting from day 3. Rapamycin or vehicle (2 mg/kg) was administrated intraperitoneally every other day starting from day 5. Tumors were harvested on day 15. (**B**) Tumor growth curve of MC38 RAC1^A159V^ tumors grown in C57BL/6 mice treated as indicated (*n* = 6 to 7 mice per group). (**C** to **H**) Frequencies of IFN-γ^+^ effector T cells among CD8^+^ T cells (C) and CD4^+^ T cells (D), TNF-α^+^ effector T cells among CD8^+^ T cells (E) and CD4^+^ T cells (F), and CD44^+^ activated T cells among CD8^+^ T cells (G), and CD4^+^ T cells (H) within the TIME of tumors in (B). Representative pseudocolor plots (left) and dot plots of cell percentage (right) are shown. (**I**) Quantification of CD8^+^ T cell (left), CD4^+^ T cell (middle) and CD11b^+^ myeloid (right) infiltration as counts per tumor weight within the TIME of tumors in (B). (**J**) Flow cytometry analysis of tumor cell surface IFNGR1 expression on tumor cells (gated as CD45^−^CD98^+^) from tumors in (B). Representative histogram (left) and MFI summary (right) are shown. Data are representative of at least two independent experiments [(B) to (J)]. Data represent means ± SD. Statistical significance determined by two-way ANOVA (B) and one-way ANOVA [(C) to (J)]. *P < 0.05, **P < 0.01, ***P < 0.001, and ****P < 0.0001.

We next sought to determine whether targeting the RAC1^A159V^ downstream mTORC1 signaling could sensitize mutant tumors to immunotherapy. We treated MC38 RAC1^A159V^ tumor-bearing mice with rapamycin, a mTORC1 inhibitor, in combination of anti-PD1 (Fig. 6A). Notably, while anti-PD1 monotherapy failed to inhibit tumor growth, the addition of a relatively low dose rapamycin (2 mg/kg) resulted in a synergistic suppression of tumor progression (Fig. 6B). The flow cytometry analysis of the TILs revealed that the frequencies of CD8^+^IFN-γ^+^, CD8^+^TNF-α^+^ effector T cells were increased by rapamycin and with a further synergistic increase observed in the combination group (Fig. 6, C and E). Although the frequency of CD4^+^TNF-α^+^ T cells remained unchanged, the CD4^+^IFN-γ^+^ effector T cells were increased by combination treatment (Fig. 6, D and F). Furthermore, the frequency of CD44^+^-activated T cells was increased by rapamycin in both CD8^+^ and CD4^+^ T cell populations (Fig. 6, G and H). A significant increase in CD8^+^ and CD4^+^ T cell and CD11b^+^ myeloid cell infiltration was observed within the combination treatment group (Fig. 6I). These results indicate that rapamycin was able to convert the immunologically cold TIME of RCA1^A159V^ tumor to hot, thereby overcoming resistance to anti-PD1.

We next tested whether the mTORC1 inhibition could reverse the observed dysregulation of tumor glycolysis, chemokine expression, and IFNGR1 by RAC1^A159V^. Rapamycin treatment increased interstitial fluid glucose concentrations, suggesting reduced glucose uptake by tumor cells and consequently increased glucose availability for infiltrating immune cells within the TIME (fig. S13A). Consistently, rapamycin treatment up-regulated the expression of glycolysis-related genes in CD8^+^ T cells and down-regulated the expression of these genes within the tumor cells (fig. S13, B and C). In line with in vitro cell culture observations, rapamycin treatment promoted chemokine production and IFNGR1 expression within tumor cells, suggesting a restoration of immune cell infiltration and tumor response to IFN-γ signaling (Fig. S13D, 6 J & S13E). These in vivo data further support a mechanism by which hyperactive mTORC1 signaling in RAC1^A159V^ tumors attenuates ICI efficacy through increased tumor glycolysis, suppressed chemokine production, and down-regulated IFNGR1 expression.

Rapamycin is a known immunosuppressor and may also affect T cell activation, differentiation, and function in the TIME (*52*, *53*). We used flow cytometry to quantify the levels of p4E-BP1, a downstream effector of mTOR signaling, within CD45^−^CD98^+^ MC38 tumor cells, CD4^+^ T cells, and CD8^+^ T cells. At our administered dosage, rapamycin selectively inhibited p4E-BP1 within the tumor cells without affecting that in the T cell populations (fig. S13, F to H), suggesting that the observed immunomodulatory effects of rapamycin are primarily mediated through its direct effects on the tumor cells while sparing T cells. Because MC38 RAC1^WT^ tumors exhibit a strong response to anti-PD1, we optimized the treatment regimen by reducing the anti-PD1 dosage to better delineate potential synergistic effects in MC38 RAC1^WT^ tumors (fig. S14A). Different from the observations of MC38 RAC1^A159V^ tumors, while rapamycin monotherapy exhibited antitumor activity by itself toward MC38 RAC1^WT^ tumors, it did not show a synergistic efficacy in combination with anti-PD1 in this context (fig. S14, B and C). This differential effect on WT versus RAC1^A159V^ tumors is likely due to the hyperactivation of mTOR found in RAC1^A159V^ tumors, which renders them more sensitive to rapamycin. The result also aligns well with the observed sphingolipid up-regulation of mTOR due to RAC1^A159V^ mutation in the tumor cells.

### RAC1^A159V/WT^ heterozygous CT26 tumors retain resistance to anti-PD1 treatment

**Fig. 7.**
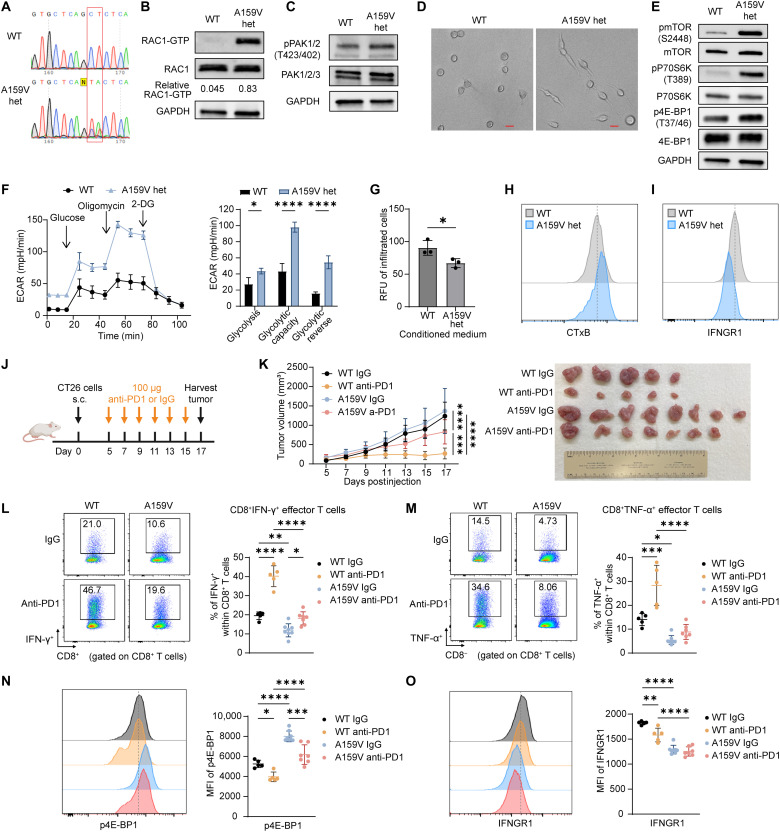
RAC1^A159V/WT^ CT26 tumors have decreased response to anti-PD1 with elevated sphingolipid-mTORC1 signaling, increased glycolysis, reduced chemotaxis, and down-regulated IFNGR1 expression. (**A**) Sanger sequencing of the RAC1 locus in CT26 RAC1^WT^ and RAC1^A159V/WT^ heterozygous mutant cells. (**B**) PAK-PBD pull-down assay to detect RAC1 activity. (**C**) Western blot analysis of RAC1 downstream phospho-PAK1/2(T423/T402). (**D**) Representative bright-field microscopy images illustrating morphology (20×; scale bars, 25 μm). (**E**) Western blot analysis of mTOR pathway–associated proteins. (**F**) Seahorse glycolysis stress test in CT26 RAC1^WT^ and RAC1^A159V/WT^ heterozygous cells (*n* = 3). (**G**) Chemotaxis assay of CD8^+^ T cells toward conditioned medium derived from CT26 RAC1^WT^ and RAC1^A159V/WT^ heterozygous cells (*n* = 3). (**H**) Flow analysis of cell membrane glycosphingolipid levels measured using Choleratoxin B (CTxB) fluorescence staining. (**I**) Flow analysis of cell surface IFNGR1 expression. (**J**) Schematic illustration of tumor inoculation and anti-PD1 administration. CT26 RAC1^WT^ and RAC1^A159V/WT^ heterozygous cells (2 million) were subcutaneously injected into BALBc/J mice on day 0. Anti-PD1 or IgG isotype control (100 μg) was administrated intraperitoneally every other day starting from day 5. Tumors were harvested on day 17. (**K**) Tumor growth curve (left) and image of tumors (right) are shown (*n* = 5 to 8 mice per group). (**L** and **M**) Frequencies of IFN-γ^+^ (L) and TNF-α^+^ (M) effector T cells among CD8^+^ T cells within the TIME of tumors in (K). Representative pseudocolor plots (left) and dot plots of cell percentage (right) are shown. (**N** and **O**) Flow cytometry analysis of p4E-BP1 and cell surface IFNGR1 expression in tumor cells (gated as CD45^−^CD98^+^) from tumors in (K). Representative histogram (left) and MFI summary (right) are shown. Data are representative of at least two independent experiments [(B) to (F)]. Data represent means ± SD. Statistical significance determined by two-way ANOVA (F), unpaired *t* test (**G**), and one-way ANOVA [(L) to (O)]. *P < 0.05, **P < 0.01, ***P < 0.001, and ****P < 0.0001.

Because most oncogene mutations in patients are heterozygous and heterozygous oncogene mutation is sufficient for tumorigenesis (*54*–*56*), we sought to determine whether the heterozygous RAC1^A159V/WT^ mutation may similarly promote an immunosuppressive TIME and confer tumor resistant to anti-PD1. To this end, we engineered the heterozygous RAC1^A159V/WT^ mutation in an additional murine colon carcinoma cell line, CT26. Sanger sequencing confirmed the presence of heterozygous RAC1^A159V/WT^ mutation, with one allele encoding WT RAC1 and the other carrying the A159V substitution (Fig. 7A). RAC1-GTP pull-down assay revealed elevated RAC1 activity in RAC1^A159V/WT^ heterozygous cells compared to WT cells (Fig. 7B). The phosphorylation of RAC1 downstream effectors pPAK1/2 was also significantly up-regulated in the RAC1^A159V/WT^ heterozygous cells (Fig. 7C), and the morphology of CT26 RAC1^A159V/WT^ heterozygous cells displayed a more spindle-like phenotype with extended filopodia compared with WT cells (Fig. 7D). Consistent with prior observations in MC38 and B16OVA cells harboring RAC1^A159V^ homozygous mutation, key components of the downstream mTOR pathway including pmTOR, pS6K, and p4E-BP1 were up-regulated in CT26 RAC1^A159V/WT^ heterozygous cells (Fig. 7E). Glycolysis stress test and chemotaxis assay demonstrated that RAC1^A159V/WT^ heterozygous cells had enhanced glycolysis and reduced chemotaxis ability to attract CD8^+^ T cells (Fig. 7, F and G). Furthermore, flow cytometry analysis identified an up-regulation of cell membrane glycosphingolipids and a down-regulation of IFNGR1 expression in the RAC1^A159V/WT^ heterozygous cells (Fig. 7, H and I). These cellular data suggest that CT26 tumor cells with heterozygous RAC1^A159V/WT^ mutation exhibit similar alterations in key signaling pathways as those found with homozygous mutation.

To evaluate the in vivo impact of the tumor-derived heterozygous RAC1^A159V/WT^ mutant on the TIME and ICI response, CT26 RAC1^WT/WT^ and heterozygous RAC1^A159V/WT^ cells were subcutaneously injected into syngeneic BALB/c mice and treated with anti-PD1 or IgG isotype control every other day after tumors became palpable (Fig. 7J). Notably, RAC1^A159V/WT^ heterozygous tumors are partially resistant to anti-PD1, as evidenced by a reduced anti-PD1 treatment efficacy compared to WT tumors (Fig. 7K). Flow cytometry analysis of TILs revealed a decrease in the frequency of CD8^+^IFN-γ^+^ effector T cells and CD8^+^TNF-α^+^ effector T cells within A159V heterozygous tumors (Fig. 7, L and M). While anti-PD1 significantly increased the frequency of effector T cell in WT tumors, its effect was markedly attenuated in RAC1^A159V/WT^ heterozygous tumors (Fig. 7, L and M). Consistent with the in vitro data, mTOR downstream p4E-BP1 was up-regulated, and cell surface IFNGR1 was down-regulated in CT26 RAC1^A159V/WT^ tumor cells in vivo (Fig. 7, N and O). However, unlike MC38 homozygous A159V tumors, CT26 RAC1^A159V/WT^ heterozygous tumors did not show a growth advantage in immunocompetent mice and were partially resistant to anti-PD1 (Fig. 7H). This is likely attributable to the dosage effect of the RAC1^A159V^ mutation. Together, these data demonstrate that RAC1^A159V/WT^ heterozygous mutation can significantly reduce anti-PD1 efficacy via similar mechanistic pathways as homozygous mutation, and it is likely conserved across different tumor models. The heterozygous model may be able to more accurately recapitulate the genomic alteration observed in patients and provide useful implications for RAC1^A159V^-mediated resistance to ICI therapy.

## DISCUSSION

**Fig. 8.**
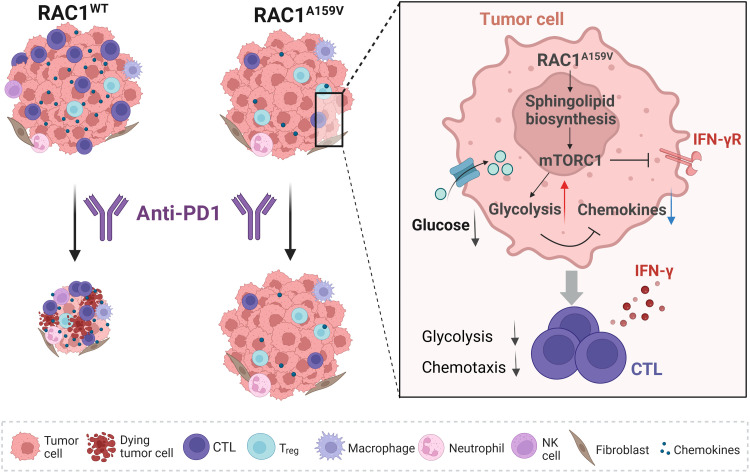
A schematic summary of the results. RAC1^A159V^ mutation renders the tumor cells resistant to anti-PD1 treatment with an immunosuppressive TIME. This is mediated by elevated sphingolipid-mTORC1 signaling leading to reduced chemokine and IFNGR1 productions and increased glycolysis.

The immunosuppressive TIME, often characterized by a high ratio of immunosuppressive cells (such as Treg cells) to antitumor effectors (such as CD8^+^ cytotoxic T cells), represents a significant barrier to the efficacy of ICIs (*7*, *8*, *57*). Hence, targeting modulators of the TIME has emerged as a promising strategy for enhancing the outcomes of cancer immunotherapy. In this study, we demonstrate that the tumor-derived RAC1^A159V^ mutation, a recurrent GOF mutation in RAC1, creates an immunosuppressive TIME, facilitating tumor evasion from immune attack. The tumors harboring the RAC1^A159V^ mutation exhibit characteristics of immunologically cold tumors, including a significant decrease in immune cell infiltration and a marked reduction in tumor-immune cell communications. RAC1^A159V^ mutant exerts its immunosuppressive effects on the TIME through the activation of mTORC1 signaling via several pathways. Specifically, up-regulated mTORC1 signaling in RAC1^A159V^ tumor cells increases tumor cell glycolysis and glucose consumption, leading to reduced glucose availability within the TIME that can compromise the antitumor function of CD8^+^ T cells. In addition, signaling through mTORC1 decreases the production of chemokines in tumor cells, which impairs the recruitment of immune cells, including CD8^+^ effector T cells. Furthermore, hyperactivated mTORC1 signaling shields RAC1^A159V^ tumor from IFN-γ immune attack via down-regulating IFNGR1 expression due to increased glycosphingolipid biosynthesis. Our results establish a causal connection between these pathways and the immunosuppressive TIME associated with ICI resistance, providing the mechanism of the immune resistance feature of RAC1^A159V^ tumors (Fig. 8).

Multiple mTOR inhibitors have been used in clinical trials for various cancer types (*58*). Among these, rapamycin (also known as sirolimus) has been approved by the US Food and Drug Administration (FDA) for cancer treatment (*59*). Besides directly inhibiting cancer cell growth, rapamycin can also inhibit CD8^+^ T cell exhaustion and promote CD8^+^ memory T cell formation to enhance immunotherapeutic efficiency (*60*, *61*). Rapamycin is also known as an immunosuppressant used to prevent rejection in organ transplantation (*62*). These distinct functions of rapamycin are dosage dependent (*63*). In this study, we found that rapamycin, at the examined low dosage, targets mTOR in tumor cells without exerting effects on the immune cells, particularly CD8^+^ T cells, sensitizing RAC1^A159V^ tumors to ICIs. This is likely because the mutant tumor cells have elevated mTORC1 activity, leading to preferential rapamycin sensitivity by the tumor cells. Consistently, similar rapamycin dosage enhances ICI efficacy in RAC1^A159V^, but not RAC1^WT^ tumors, allowing the usage of lower rapamycin dosage for RAC1^A159V^ cancer immunotherapy with reduced drug side effects.

RAC1^A159V^ is the second most common RAC1 mutation following RAC1^P29S^. However, unlike RAC1^P29S^, which is primarily found in melanoma, RAC1^A159V^ exhibits a broader tissue distribution, occurring across various cancer types including colon cancer, head and neck cancer, lung cancer, and melanoma (*14*). Despite its prevalence, the RAC1^A159V^ mutation remains poorly studied, particularly its impact on the TIME. Available patient data regarding RAC1^A159V^-mutated tumors in large-scale genomic datasets such as The Cancer Genome Atlas are limited. A single available RAC1^A159V^ patient case with documented ICI response exhibits resistance (sample ID: MEL-IPI_Pat138-Tumor-SM-7A155); further clinical studies of RAC1^A159V^ cases are warranted to test the validity of our animal model prediction of immune resistance to ICI therapies.

We have used CRISPR-Cas9–mediated endogenous gene editing to generate the RAC1^A159V^ homozygous and heterozygous mutation in three different tumor cell models and investigated their effects on the TIME. This approach ensures that the mutation is expressed at physiological levels within RAC1 native genomic context, circumventing limitations associated with various overexpression systems. Given that Rho GTPase signaling is essential for regulating critical cellular processes such as cell survival, proliferation, and differentiation (*64*), cells require a balance of Rho GTPase signaling to maintain normal physiological activity. For example, the hyperactivation of RAC1 has been shown to induce cell death (*65*). We found the overexpression of RAC1^P29S^, another GOF RAC1 mutant, via lentiviral transduction, results in cellular stress and dysfunction (unpublished data), suggesting that varying levels of RAC1 activity may elicit distinct downstream signaling outcomes.

A recent report studying RAC1^P29S^ in YUMM1.7 melanoma cells found that this RAC1 mutation sensitizes the tumor cells to anti-PD1 therapy (*30*), distinct from our findings here. Aside from the potential difference between our endogenous mutation model and the overexpression system used in the RAC1^P29S^ study, it is possible that RAC1^P29S^ and RAC1^A159V^ display different signaling strength/duration that may affect downstream signaling differently (*25*). Similarly, differential signaling outcomes were also observed in other small GTPases. For example, RhoA^Y42C^ has increased binding to ROCK and decreased binding to Rhotekin, whereas RhoA^L57V^ has unaltered binding to diverse effectors (*66*). Then, increased AKT activation was observed in KRAS^G12D^ but not KRAS^G12C^ cells (*67*), and KRAS^G12C^ and KRAS^G12D^ showed distinct responses to ICIs. The KRAS^G12C^ tumors displayed better response to anti–PD1/PD-L1 than KRAS^WT^ tumors, whereas the KRAS^G12D^ tumors were more resistant (*68*–*70*).

Overall, our studies help elucidate the multifaceted role of RAC1^A159V^ in shaping an immunosuppressive TIME and provide a mechanistic insight into its contribution to immune evasion. The findings suggest that RAC1^A159V^ can be a potential predictor for ICI response and present a strategy for RAC1^A159V^ therapy by combining ICIs with a targeted inhibition of RAC1 signaling to overcome ICI resistance.

## MATERIALS AND METHODS

### Cells

MC38 mouse colon adenocarcinoma cells were purchased from Kerafast (catalog no. ENH204-FP). B16OVA MO4 mouse melanoma cells were purchased from Sigma-Aldrich (catalog no. SCC420). CT26 cells were purchased from American Type Culture Collection (catalog no. CRL-2638). MC38 cells were grown in Dulbecco’s modified Eagle’s medium (DMEM) medium supplemented with 10% fetal bovine serum (FBS) and 1% penicillin/streptomycin. B16OVA MO4 cells were grown in RPMI 1640 medium supplemented with 10% FBS, 1% penicillin/streptomycin, 1x non-essential amino acids, and 10 mM Hepes. CT26 cells were grown in RPMI 1640 medium supplemented with 10% FBS and 1% penicillin/streptomycin. All cells were grown in a humidified incubator of 5% CO_2_ at 37 °C. All cell lines were tested for mycoplasma using the Mycoplasma Detection Kit (Biological Industries USA).

### CRISPR-Cas9–mediated gene editing

MC38 or B16OVA RAC1^A159V^-mutated cells were generated with CRISPR-Cas9–mediated knock-in technology. Single guide RNA (sgRNA) sequence was designed using a public online tool (http://crispor.tefor.net). Alt-R sgRNA, Alt-R HDR Donor Oligos, and Alt-R S.p. Cas9-GFP nuclease were ordered from Integrated DNA Technologies (IDT). A 5 μg of Cas9 and 1.2 μg of sgRNA were incubated at 37°C for 10 min to form ribonucleoprotein (RNP) complex. Subsequently, the RNP complex was combined with 100 pmol of donor DNA and electroporated into 200,000 cells using a 4D-Nucleofector X Unit (Lonza, Cologne, Germany). The specific conditions were as follows: SE kit and program EN-113 for MC38 cells, SF kit and program CU-137 for B16OVA cells, and SE kit and program DS-156 for CT26 cells. Following electroporation, cells were maintained in 1-ml medium with 1.5 μl of HDR enhancer V2 (IDT) for 24 hours. Then, the medium was replaced with fresh medium without HDR enhancer. Forty-eight hours postelectroporation, cells were seeded in 96-well plate with single cell per well by fluorescence-activated cell sorting (FACS) sorting (BD FACSAria Fusion Cell Sorter), and expanded until colonies were ready to be picked. Picked cell colonies were extracted genomic DNA with PureLink Genomic DNA Mini Kit (Thermo Fisher Scientific). Fragment with A159V mutation was enriched by PCR, and then the mutation was confirmed by Sanger sequencing:

A159V sgRNA: TGCTCAGCTCTCACACAGCG, A159V donor DNA: GAGTGCTCAGTACTCACtCAGCGAGGACTCAAGACAGTG, and A159V primers:

forward: 5′ CCACCTGAGAGAGGGAAGT 3′; reverse: 5′ CAAATGCGAAGGCTCGCTG 3′.

### In vitro drug treatment

A total of 1 × 10^5^ cells were seeded in each well of six-well plate and cultured overnight, then drug was added into the cell culture medium, and cells were treated for 24 hours. Rapamycin was purchased from MedChemExpress (catalog no. HY-10219), 2 μM was used for RT-qPCR detection of glycolysis-related gene and chemokines, and 5 μM was used for flow cytometry analysis of cell surface IFNGR1 expression. 2-DG was purchased from Sigma-Aldrich (catalog no. D8375), and 2 mM was used. Eliglustat was purchased from MedChemExpress (catalog no. HY-14885), and 20 μM was used.

### Animal models

C57BL/6, BALB/cJ, and NSG (NOD-scid IL2Rgamma^null^) female or male mice (6 to 8 weeks old) were purchased from Jackson Laboratories (Bar Harbor, ME, USA). No significant sex-dependent differences were found in the experiments reported. All mice were housed under specific pathogen–free conditions in the animal facility at the Cincinnati Children’s Hospital Research Foundation in compliance with the CCHMC Institutional Animal Care and Use Committee (CCHMC IACUC, approved no. IACUC2023-0016). A suspension of 2 × 10^6^ MC38 or CT26 cells in 100 μl of PBS was mixed with 100 μl of Growth Factor Reduced Matrigel (Corning, catalog no. 354230) and subcutaneously inoculated. A suspension of 1 × 10^6^ B16OVA cells in 50 μl of PBS was intradermally inoculated. Anti-PD1 (RMP1-14, Bio X Cell, catalog no. BE0146) or IgG isotype control (Bio X Cell, catalog no. BE0089) in PBS were administered intraperitoneally at 100 μg per body. Rapamycin (MedChem Express, catalog no. HY-10219) was administered intraperitoneally at 2 mg/kg dissolved in 2% dimethyl sulfoxide, 30% polyethylene glycol 300 (MedChem Express, catalog no. HY-Y0873), and 5% Tween 80 (Sigma-Aldrich, P1754). Mice were randomized at the first day of treatment to control or drug in an unblinded manner. Tumor volume was monitored every 2 days and was calculated as length × width^2^ × 0.5. All mice were euthanized when the biggest tumor volume was >1800 mm^3^, and tumors were harvested.

### Tumor dissociation and cell isolation

Tumors were minced into small fragments and treated with collagenase IV (0.8 mg/ml; Sigma-Aldrich, catalog no. C5138) for 1 hour at 37°C under agitation. The digested tumor tissue was then filtered through a 70-μm cell strainer and centrifuged at 300*g* at 4°C for 5 min. A subset of the cell population (2 × 10^7^ cells) was processed for tumor cell isolation using the Tumor Cell Isolation Kit (Miltenyi Biotec, catalog no. 130-110-187), following the manufacturer’s protocol. The isolated tumor cells were used for western blotting, RT-qPCR and RNA sequencing. The remaining cells were resuspended in 4 mL of 40% percoll and slowly layered over 6 mL of 80% percoll in a 15 mL falcon tube. The falcon tube was then centrifuged at 2000 rpm at 4 °C for 20 min, stopping without brakes. Cells at the interface containing immune cells were carefully removed, washed twice, and resuspended in PBS. The resulting single cell suspension was subsequently used for flow cytometry analysis or for isolating tumor-infiltrating CD8^+^ T cells. The CD8^+^ T cell isolation was performed using CD8a (Ly-2) MicroBeads (Miltenyi Biotec, catalog no. 130-117-044) according to the manufacturer’s protocol. The isolated CD8^+^ T cells were used for RT-qPCR and chemotaxis assay.

### Flow cytometry assays

Cell surface proteins were stained for 30 min at 4 °C. Intracellular proteins were stained for 30 min at 4°C after permeabilization and fixation with BD Cytofix/Cytoperm Plus (BD Biosciences, catalog no. 555028) as manufacturer’s protocol. For intracellular cytokine detection, cells were stimulated for 4 hours with phorbol 12-myristate 13-acetate (25 ng/ml)/ionomycin (1 mg/ml) (Sigma-Aldrich) and GolgiPlug reagent (1 μl/ml) (BD Biosciences), and then the stained cells were subjected to flow cytometry analysis. The following antibodies were used: CD45 peridinin-chlorophyll-protein (catalog no. 557235), CD8a fluorescein isothiocyanate (catalog no. 553031), IFN-γ allophycocyanin (APC) (catalog no. 554413), CD11b APC (catalog no. 553312), CD206 Alexa Fluor 488 (catalog no. 568807), and IFNGR1 BV650 (catalog no. 740498) from BD Biosciences; CD4 eFluor450 (catalog no. 48–0341-82), FOXP3 phycoerythrin (PE) (catalog no. 12-5773-82), TIM3 PE/Cy7 (catalog no. 25-5870-82), TNF-α PE/Cy7 (catalog no. 25-7321-82), CD86 PE-Cy7 (catalog no. 25-0862-82), and CD206 eFlour450 (catalog no. 48-2061-80) from eBiosciences; CD44 PE (catalog no. 103007), F4/80 PE (catalog no. 123110), Ly-6G/Ly-6C APC/Cy7 (catalog no. 108424), and CD98 PE (catalog no. 128207) from BioLegend; p4E-BP1(Thr^37/46^) Alexa Fluor 488 (catalog no. 2846S) from Cell Signaling Technology; and CTxB Alexa Fluor 488 (catalog no. C34775) from Invitrogen. The cells were analyzed by BD LSRFortessa flow cytometer, and the data were analyzed with BD FACSDiva and FlowJo_v10.7.0.

### Western blotting

Total proteins were extracted with radioimmunoprecipitation assay buffer (Santa Cruz Biotechnology, catalog no. sc-24948), and concentrations of the protein samples were determined by Bio-Rad Protein Assay Kit II (catalog no. 5000002). A 15 μg of protein from each sample was run in a 4 to 15% SDS polyacrylamide gel (Bio-Rad) and transferred onto nitrocellulose membranes. The membranes were blocked in 5% milk in tris-buffered saline–Tween 20 (TBST) for 1 hour. Blocked membranes were incubated overnight with primary antibodies in 5% bovine serum albumin in TBST. After washing and incubating with the appropriate horseradish peroxidase (HRP)–conjugated secondary antibody, protein signals were detected using ECL Western blot detection reagents (GE Healthcare and Thermo Fisher Scientific). Images were taken by the ChemiDoc Touch Imaging System (Bio-Rad). The following antibodies were used (obtained from Cell Signaling Technology unless otherwise indicated): PAK1/2/3 (catalog no. 2604S), phospho-PAK1(Thr^423^)/2(Thr^402^) (catalog no. 2601S), ERK1/2 (catalog no. 4695S), phospho-ERK1/2 (Thr^202^/Tyr^204^) (catalog no. 8544S), mTOR (catalog no. 2983S), phospho-mTOR (Ser^2448^) (catalog no. 5536S), p70S6K (catalog no. 9202S), phospho-p70S6K (Thr^389^) (catalog no. 9205S), 4E-BP1 (catalog no. 9466S), p4E-BP1 (Thr^37/46^) (catalog no. 2855S), vinculin (catalog no. 13901S), glyceraldehyde-3-phosphate dehydrogenase (Proteintech, catalog no. HRP60004), β-actin (catalog no. 5125S), and HRP-linked anti-rabbit IgG (catalog no. 7074S). The band intensity was quantified by Image Lab and Photoshop.

### Endogenous RAC1 activity assay

MC38 or B16OVA cells were cultured to a confluency of 80% in 10-cm dishes. Cells were lysed in a buffer containing 20 mM tris-HCl (pH 7.6), 100 mM NaCl, 10 mM MgCl_2_, 1% Triton X-100, 2 mM NaF, and protease inhibitors [2 mM phenylmethylsulfonyl fluoride, leupeptin (10 μg/ml), aprotinin (10 μg/ml)]. Concentrations of the protein samples were determined by Bio-Rad Protein Assay Kit II (catalog no. 5000002). The GTP-bound RAC1 in the lysate was detected by GST-PAK1 effector domain pull-down method as reported (*71*). Briefly, the cell lysate was incubated at 4°C for 1 hour with GST-PAK1 beads. After washing, the bead-bound RAC1-GTP proteins were quantified by Western blotting with anti-RAC1 (Sigma-Aldrich, catalog no. 05-389) primary antibody and HRP-linked anti-rabbit IgG (Cell Signaling Technology, catalog no. 7076S). To compare the level of the active RAC1, the amount of RAC1-GTP protein was normalized to the total amount of RAC1 in cell lysates.

### F-actin staining

Actin filaments were fluorescently stained using Acti-stain 555 phalloidin (Cytoskeleton, catalog no. PHDH1) following manufacturer’s protocol. Briefly, 2.5 × 10^4^ cells were seeded per well in eight-well chamber slides (Ibidi, catalog no. 80826) and cultured overnight to allow cell attachment. Cells were washed with PBS and fixed in 4% paraformaldehyde in PBS for 10 min and permeabilized with 0.5% Triton X-100 in PBS for 5 min. Then, cells were stained with 100 nM Acti-stain 555 phalloidin in dark for 30 min and counterstained with 100 nM 4′,6-diamidino-2-phenylindole. Then, slides were washed with PBS, mounted with Prolong Gold Antifade Mountant (Thermo Fisher Scientific, catalog no. P36930), and allowed to dry overnight in dark. Images were acquired with Nikon Eclipse Ti confocal microscope and 60× oil immersion objective.

### Cell proliferation assay

Cell proliferation was evaluated by the CellTiter 96 AQueous One Solution Cell Proliferation Assay (MTS) (Promega) every 24 hours according to the manufacturer’s instructions. A total of 1 × 10^3^ cells were initially seeded per well into a 96-well plate as 200 μl of cell suspensions and cultured for 24 hours. Then, 20 μl per well of CellTiter 96 AQueous One Solution reagent was added. After 2-hour incubation in humidified at 37°C with 5% CO_2_ atmosphere, absorbance at 490 nm was measured using a SpectraMax I3 microplate reader (Molecular Devices, Sunnyvale, CA). Three replicate wells per time point were used to obtain measures of cell proliferation.

### Cell imaging

MC38 RAC1^WT^ and RAC1^A159V^ cells were seeded in a 12-well plate at a density of 2 × 10^5^ cells per well. The following day, once cells were adherent and confluent, a 20-μl pipette tip was used to scratch a vertical line across the monolayer of cells. Detached cells were removed by gently washing cell monolayer. After 24 hours, cells at the leading edge were imaged. CT26 RAC1^WT^ and RAC1^A159V/WT^ heterozygous cells were seeded in a six-well plate at a density of 1 × 10^5^ cells per well. The following day, cell morphology was imaged. All images were acquired with a Widefield Nikon Ti2 inverted SpectraX microscope and a 20× objective under bright-field settings.

### Tumor interstitial fluid collection and glucose concentration measurement

Tumor interstitial fluid (TIF) was isolated from tumors using a previously described centrifugal method (*72*). Briefly, tumor bearing mice were euthanized and tumors were rapidly dissected from the mice. The tumors were then put onto 20-μm Nylon Membrane Filter (Sigma-Aldrich, catalog no. NY2004700) affixed to the 50-ml conical tubes, and centrifuged at 4°C for 10 min at 106*g*. Flow-through interstitial fluid was flash-frozen and stored at −80°C before analysis. The concentration of glucose was quantified by Glucose Colorimetric Detection Kit (Thermo Fisher Scientific, catalog no. EIAGLUC).

### RT-qPCR analyses

RNA was extracted using the miRNeasy Micro Kit (QIAGEN, catalog no. 217084), cDNA was generated using High-Capacity cDNA Reverse Transcription Kit (Applied Biosystems, catalog no. 4368814), and real-time qRT-PCR was performed with predesigned Taqman Gene Expression assays (Thermo Fisher Scientific) using the StepOnePlus Real-Time PCR Systems (Applied Biosystems). Gene expression changes relative to the expression of actb RNA, which was used as a housekeeping gene, were calculated using the ΔΔCt method. Assay IDs are: Slc2a1 (Mm00441480_m1), Slc16a3 (Mm00446102_m1), Hif1a (Mm00468869_m1), Aldoc (Mm01298116_g1), Hk2 (Mm00443385_m1), Ccl2 (Mm00441242_m1), Ccl5 (Mm01302427_m1), Ccl7 (Mm00443113_m1), Ccl8 (Mm01297183_m1), Ccl11 (Mm00441238_m1), Ccl19 (Mm00839967_g1), Cxcl10 (Mm00445235_m1), Ifngr1 (Mm00599890_m1), St3gal1 (Mm00501493_m1), St3gal2 (Mm00486123_m1), B4galt5 (Mm00480147_m1), Sptlc2 (Mm00448871_m1), Ugcg (Mm00495925_m1), and Actb (Mm02619580_g1).

### Chemotaxis assay

Chemotaxis was assessed using the CytoSelect 96-well cell migration assay kit (Cell Biolabs, catalog no. CBA-104). MC38 RAC1^WT^ and RAC1^A159V^ were cultured for 2 days, and the conditioned medium was collected separately. CD8^+^ T cells isolated from tumors were cultured overnight in RPMI 1640 medium supplemented with 5% FBS, 1% penicillin/streptomycin, 1× non-essential amino acids, 10 mM Hepes, 2 mM l-glutamine and murine IL-2 (20 ng/ml) in humidified at 37°C and 5% CO_2_ incubator. Then, 0.5 × 10^6^ CD8^+^ T cells per well were seeded in 96-well migration plate in 100-μl medium without IL-2. A 150-μl conditioned medium was added in the bottom chamber as the chemoattractant. The transwells were incubated for 5 hours at 37°C and 5% CO_2_. The migrated CD8^+^ T cells were measured by the quantification of fluorescence following the manufacturer’s protocol.

### Glycolysis stress test

MC38, B16OVA, or CT26 cells were seeded at a total number of 5 × 10^4^ cells per well in Seahorse XF DMEM Medium pH 7.4 (Agilent, catalog no. 103575-100) supplemented with 2 mM l-glutamine, with or without 2 μM rapamycin (MedChemExpress, catalog no. HY-10219) 10 hours before the beginning of the experiment. The extracellular acidification rate was monitored in a Seahorse XF Pro analyzer (Agilent) using the glycolysis stress test protocol of 3-min mix, 3-min wait, and 3-min measure. Cells were challenged with 25 mM glucose (Sigma-Aldrich, catalog no. D8066), 10 μM oligomycin (Cayman, catalog no. 11341), and 100 mM 2-DG (Sigma-Aldrich, catalog no. D8375), allowing three measurements after each injection. Data were analyzed in Agilent Wave software v.2.6.

### Single cell RNA-seq

C57BL/6 mice with MC38 tumors were euthanized on day 13 after tumor implantation. Single-cell suspensions prepared from the whole tumors were used to separate CD45^+^ and CD45^−^ cells by CD45 (TIL) MicroBeads (Miltenyi Biotec, catalog no. 130-110-618). CD45^+^ and CD45^−^ cells were then mixed at a 2:1 ratio and were loaded on a 10X Chromium Controller (10x Genomics) to generate nanoliter-scale gel beads-in-emulsions (GEMs). A 3′ Gene Expression Library was prepared for scRNA-seq by the DNA Genotyping and Sequencing Core of the Cincinnati Children’s Hospital Medical Center, using “Chromium Single Cell 3′ Reagent Kits v3.1” by 10x Genomics. Then, the GEMs were used to generate barcoded, full-length cDNA through reverse transcription reactions. Next, the barcoded, full-length cDNA was used for library construction via fragmentation, end repair, A-tailing, ligation to an index adaptor, and amplification by PCR. The final libraries were sequenced on the NovaSeq X Plus Sequencer.

Resulting fastqs were processed through the CellRanger pipeline v7.2 using 10X Genomics’ mm10-2020-A reference genome, with the setting “include introns” set to true. Background reads were removed using decontX from the celda package using the filtered barcodes as the cells to keep and the remaining cells in the raw barcode matrix as the background (*73*). Doublets were minimized using DoubletFinder (*74*). The R v4.4 library Seurat v4.4 (*75*) was used for cell type clustering and marker gene identification. Cells expressing >500 genes were retained for downstream analysis. Each sample was normalized SCTransform v2 using the glmGamPoi method, and the number of RNA molecules per cell was regressed out. Samples were integrated with common anchor genes using the Canonical Correlation Analysis method to minimize sample to sample variation. Cell clusters were determined by the Louvain algorithm using a resolution of 0.4. Uniform Manifold Approximation and Projection dimension reduction was done using the first twenty principal components. Marker genes for each cell type were calculated using the Wilcoxon Rank Sum test returning only genes that are present in a minimum of 25% of the analyzed cluster and have a minimum differential cell expression of 30%. T-cells were further subdivided into subtypes by reperforming the above methods on a subset of the data only containing the t-cells. Cell–cell interactions were determined using CellChat and Liana (*76*, *77*).

The scRNA-seq data generated in this study were deposited at the Gene Expression Omnibus under accession number GSE298274.

### Bulk RNA-seq of tumor cells

C57BL/6 mice with MC38 tumors were etuhanized on day 13 after tumor implantation, and tumor cells were isolated by the Tumor Cell Isolation Kit (Miltenyi Biotec, catalog no. 130-110-187). Total RNA was extracted with miRNeasy Micro Kit (QIAGEN, catalog no. 217084) according to the manufacturer’s instructions. The quality of RNA was examined with Agilent BioAnalyzer. RNA-seq libraries were prepared using Illumina RNA-seq Library Preparation Kit, in which cDNA was prepared from polyA-selected RNA.

The prepared RNA-seq libraries underwent next-generation sequencing of 100 base pairs from both ends (paired-end reads) and 20 M reads per sample with NovaSeq 6000 platform (Illumina, San Diego, CA).

Reads were aligned to the mm10 genome using STAR, and the resulting sam files were converted to bam files using Samtools (*78*). Aligned reads were counted with the R library GenomicAlignments’ function summarizeOverlaps to generate a reads per gene counts matrix for each sample (*79*). Differential gene expression was determined using DESeq2 (*80*). The bulk RNA-seq data generated in this study were deposited at the Gene Expression Omnibus under accession number GSE298273.

### Statistics

All statistical analyses were performed using Prism 10 (GraphPad Software). Statistical analyses between two groups were performed by using the unpaired *t* test. Statistical analyses among more than two groups were performed by using one-way ANOVA or two-way ANOVA test. *P* values < 0.05 were considered statistically significant. Data represent means ± SD.
