# Supplementary material for: Tumor-derived RAC1A159V mutation promotes an immunosuppressive microenvironment that represses response to immune checkpoint inhibitor
Source: Sci Adv. 2025 Oct 29;11(44):eaea1212. doi: 10.1126/sciadv.aea1212 (PMC12571076; doi:10.1126/sciadv.aea1212)
Supplement: Supplementary file 1 — Figs. S1 to S14 [file sciadv.aea1212_sm.pdf]

Science Advances
AAAS

Supplementary Materials for

Tumor-derived RAC1^{A159V} mutation promotes an immunosuppressive microenvironment that represses response to immune checkpoint inhibitor

Mingjun Cai *et al.*

Corresponding author: Yi Zheng, yi.zheng@cchmc.org

Sci. Adv. **11**, eaea1212 (2025)
DOI: 10.1126/sciadv.aea1212

This PDF file includes:

Figs. S1 to S14

Fig. S1.

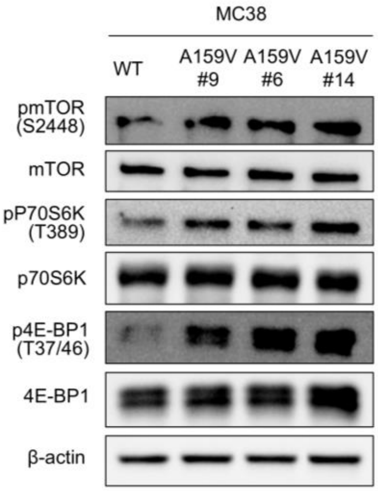

Fig. S1. Western blot analysis of mTOR pathway-associated proteins in three different MC38 RAC1^{A159V} homozygous mutant cell clones.

Fig. S2.

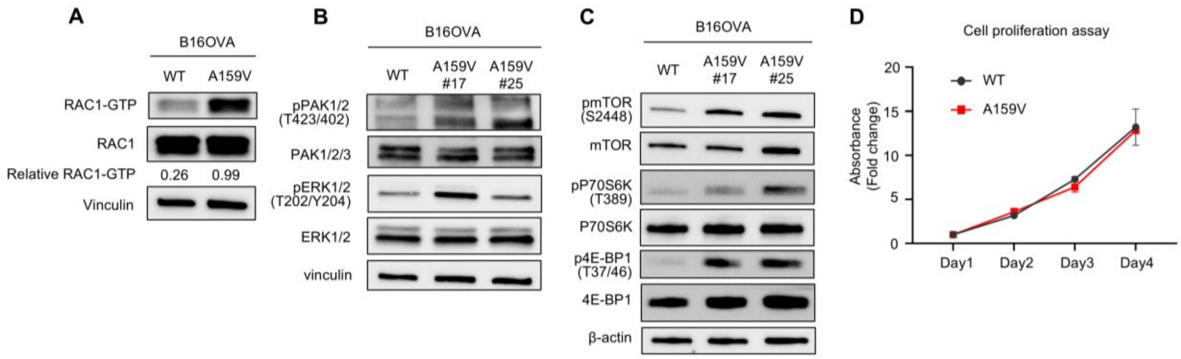

Fig. S2. Characterization of RAC1^{A159V} homozygous mutation in B16OVA melanoma cells.
(**A**) PAK-PBD pull-down assay to detect RAC1 activity of B16OVA RAC1^{WT} and RAC1^{A159V} homozygous mutant cells.
(**B**) Western blot analysis of RAC1 downstream phospho-PAK1/2 (T423/402) and phospho-ERK1/2 (T202/Y204) in B16OVA RAC1^{WT} and two different RAC1^{A159V} cell clones.
(**C**) Western blot analysis of mTOR pathway-associated proteins in B16OVA RAC1^{WT} and two different RAC1^{A159V} cell clones. Data are representative of two independent experiments.
(**D**) Cell proliferation of B16OVA RAC1^{WT} and RAC1^{A159V} cells.

Fig. S3.

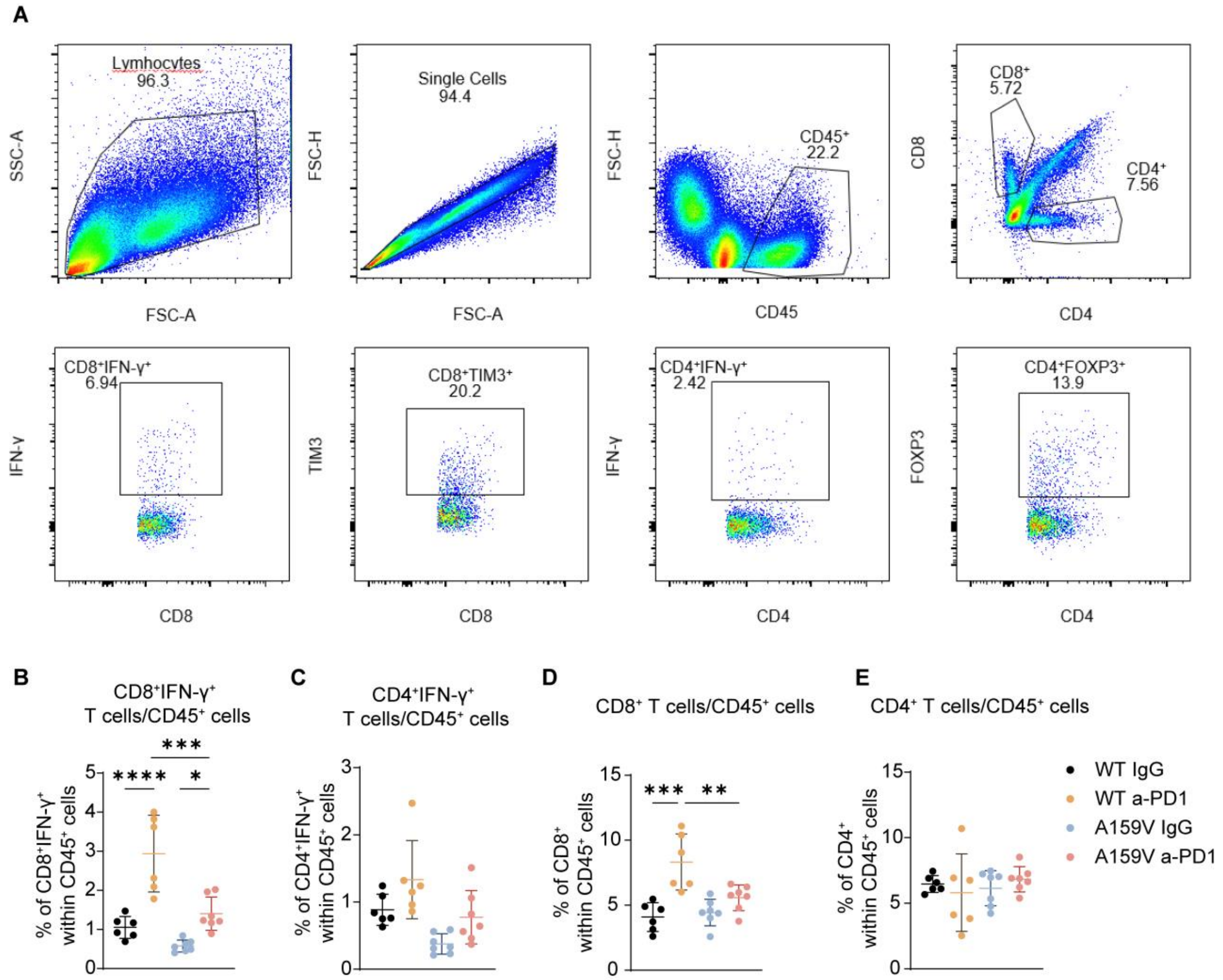

Fig. S3. Flow cytometry analyses of tumor-infiltrating T cells.
(**A**) Flow cytometry gating strategy for T cell analysis within the TIME.
(**B-E**) Frequencies of $CD8^+IFN\text{-}\gamma^+$ effector T cells (B), $CD4^+IFN\text{-}\gamma^+$ effector T cells (C), $CD8^+$ T cells (D) and $CD4^+$ T cells (E) among $CD45^+$ cells within the TIME of MC38 tumors (n=6-7 mice per group).
Data are representative of two independent experiments (**B-E**). Data represent mean ± s.d. Statistical significance determined by one-way ANOVA. **P* < 0.05; ***P* < 0.01; ****P* < 0.001; *****P* < 0.0001.

Fig. S4.

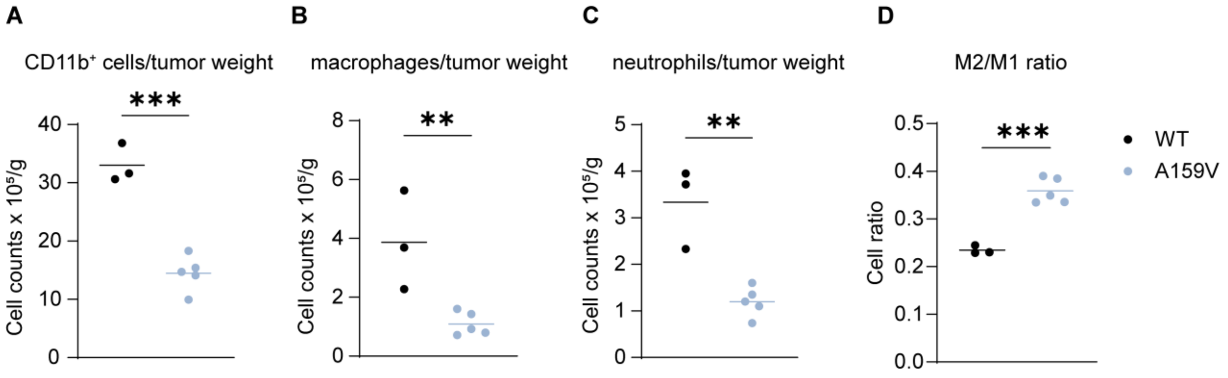

Fig. S4. Flow cytometry analyses of tumor-infiltrating myeloid cells.
(**A**-**C**) Quantification of $CD11b^+$ myeloid cell (A), $CD11b^+F4/80^+$ macrophage (B), and $CD11b^+Ly\text{-}6G/Ly\text{-}6C^+$ neutrophil (C) infiltration as counts per tumor weight within the TIME of MC38 tumors (n=3-5 mice per group).
(**D**) The ratio of M2 ($CD206^+$) to M1 ($CD86^+$) macrophages within the TIME of MC38 tumors (n=3-5 mice per group).
Data are representative of two independent experiments (**A-D**). Data represent mean ± s.d. Statistical significance determined by unpaired t-test. $**P < 0.01$; $***P < 0.001$.

Fig. S5.

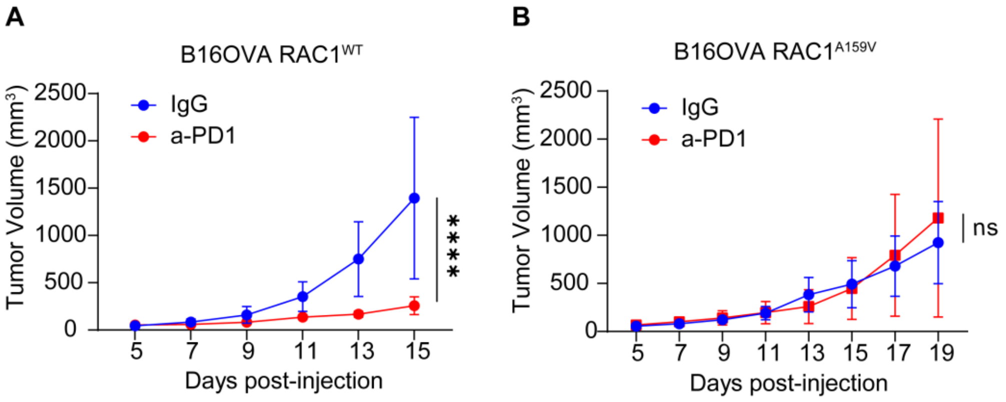

Fig. S5. B16OVA RAC1^{A159V} tumors are resistant to anti-PD1.

(**A-B**) B16OVA MO4 RAC1^{WT} and RAC1^{A159V} cells (1 million) were intradermally injected into C57BL/6 mice on day 0. Anti-PD1 or IgG isotype control (100 μg) was administrated intraperitoneally every two days starting from day 3. Tumor growth curve of RAC1^{WT} (A) and RAC1^{A159V} (B) tumors are shown (n=7 mice per group).

Data represent mean ± s.d. Statistical significance determined by two-way ANOVA. ****$P < 0.0001$.

Fig. S6.

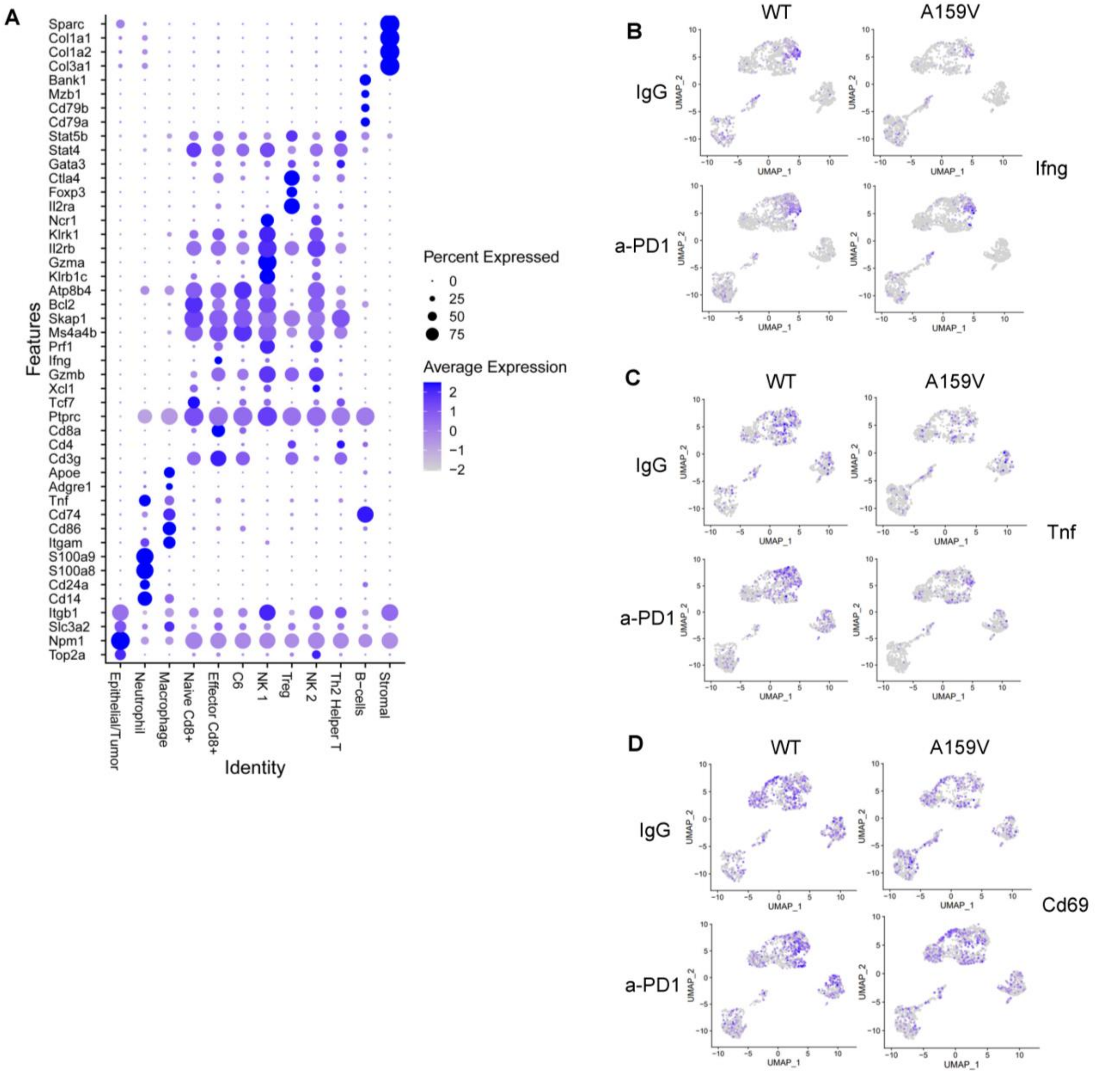

Fig. S6. Gene expressions by scRNA-seq analyses of MC38 RAC1^{WT} and RAC1^{A159V} tumors.
(**A**) Differentially expressed genes used to annotate clusters of sequenced cells.
(**B-D**) UMAP visualization of Ifng (B), Tnf (C) and Cd69 (D) expression in T cells from MC38 RAC1^{WT} and RAC1^{A159V} tumors treated with IgG or anti-PD1.

Fig. S7.

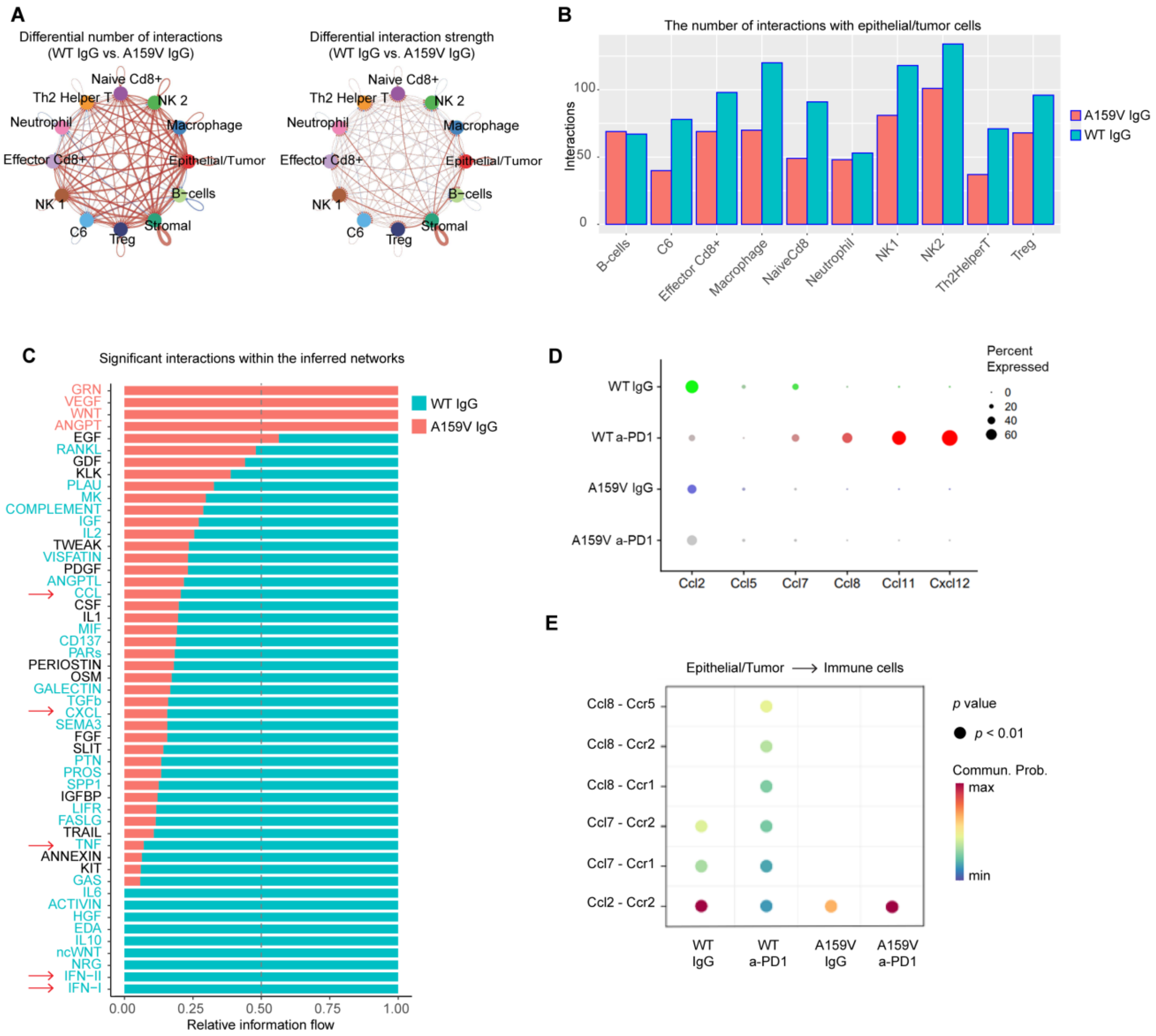

Fig. S7. RAC1^{A159V} tumor cells have decreased interaction with immune cells including reduced chemotaxis compared with RAC1^{WT} tumors.

(**A**) Circos plots of differential number of interactions (left) and differential interaction strength (right) among all cell clusters. Red lines represent increased interactions and higher interaction intensity in WT IgG tumors, while blue lines indicate increased interactions and higher intensity in A159V IgG tumors. Thicker lines indicate higher number of interactions or greater strength.

(**B**) The number of interactions between tumor cell cluster and each immune cell cluster.

(**C**) Relative information flow of significant interactions among all cell types.

(**D**) Percent of tumor cells with selected chemokine expression.

(**E**) Selected ligand-receptor chemotaxis interactions secreted from tumor cells to immune cells.

Fig. S8.

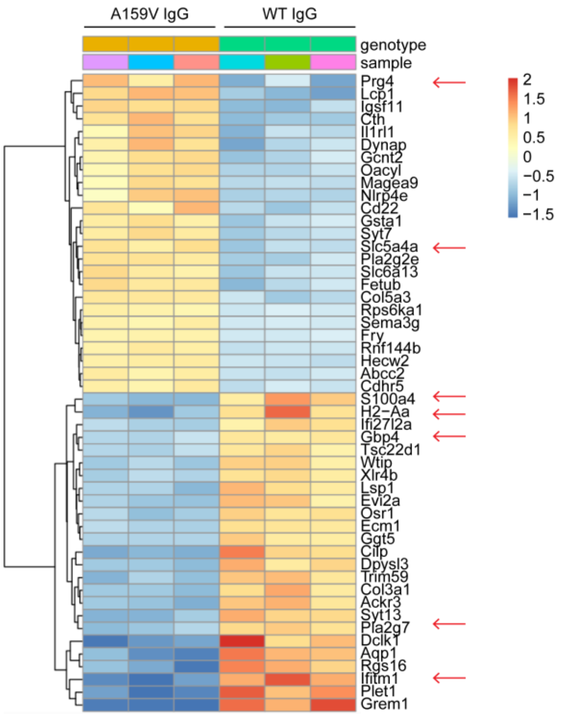

Fig. S8. Heatmap of the top 25 upregulated and downregulated genes in A159V IgG tumor cells versus WT IgG tumor cells.

Fig. S9.

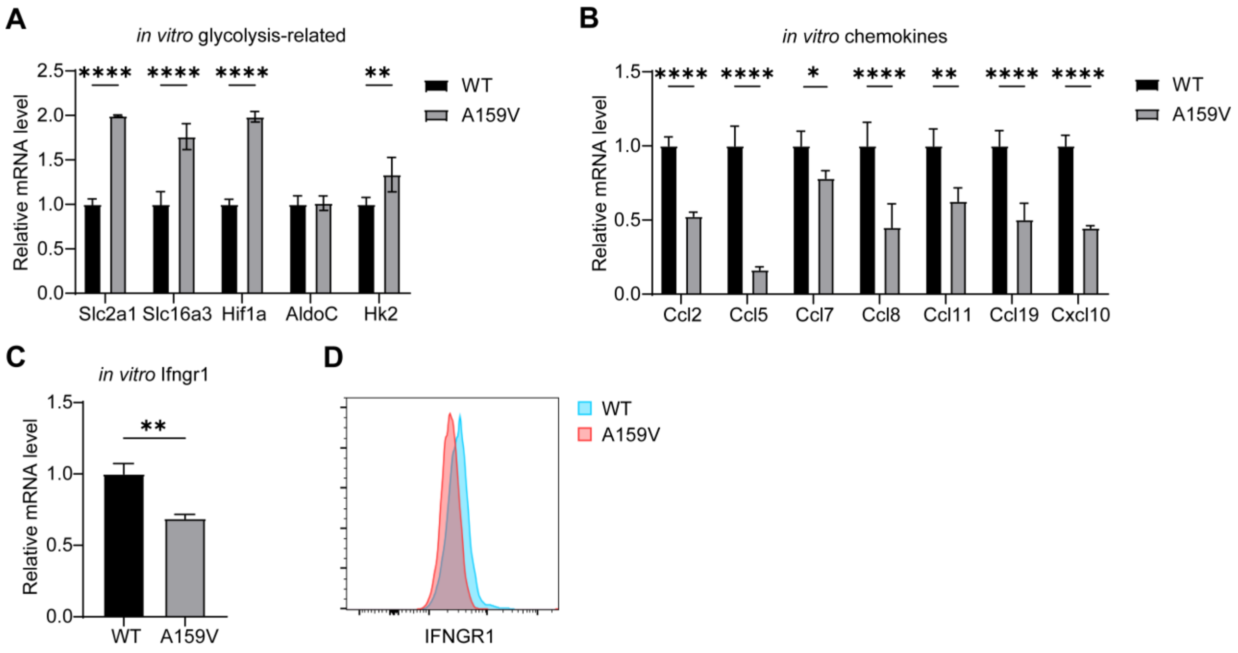

Fig. S9. RAC1^{A159V} mutation increases glycolysis and decreases chemokine and IFNGR1 expressions in tumor cells *in vitro*

(**A**-**C**) RT-qPCR analysis of selected gene expression in cultured tumor cells (n=3). (A) Glycolysis-related genes, (B) chemokines, (C) Ifngr1.

(**D**) Flow cytometry analysis of cell surface IFNGR1 expression. Representative histogram is shown.

Data are representative of three independent experiments (**C-D**). Data represent mean ± s.d. Statistical significance determined by two-way ANOVA (**A**, **B**) and unpaired t-test (**C**). *$P < 0.05$; **$P < 0.01$; ***$P < 0.001$; ****$P < 0.0001$.

Fig. S10.

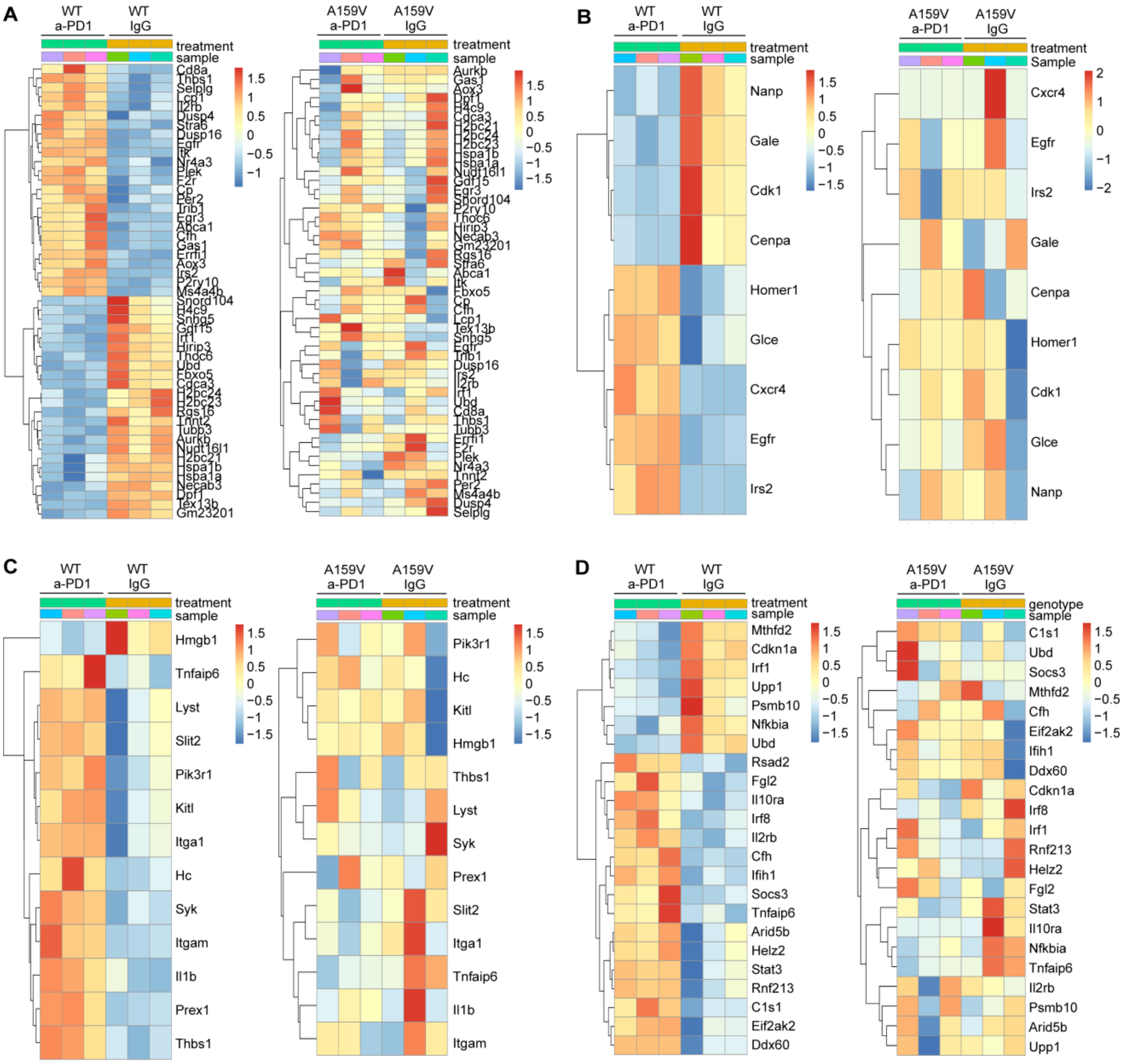

Fig. S10. Anti-PD1 treatment alters gene expression profiles in RAC1^{WT} tumors but not in RAC1^{A159V} tumors.

(**A**) Heatmap of the top 25 upregulated and downregulated genes altered by anti-PD1 in WT tumor cells (left) and the expression profile of the same genes in A159V tumor cells treated with IgG or anti-PD1 (right).

(**B**) Heatmap of the genes in the HALLMARK_GLYCOLYSIS pathway altered by anti-PD1 in WT tumor cells (left) and the expression profile of the same genes in A159V tumor cells treated with IgG or anti-PD1 (right).

(**C**) Heatmap of the genes in the GOBP_MYELOID_LEUKOCYTE_MIGRATION and GOBP_MONOCYTE_CHEMOTAXIS pathways altered by anti-PD1 in WT tumor cells (left)

and the expression profile of the same genes in A159V tumor cells treated with IgG or anti-PD1 (right).

(**D**) Heatmap of the genes in the HALLMARK_INTERFERON_GAMMA_RESPONSE, GOBP_RESPONSE_TO_TYPE_II_INTERFERON and GOBP_CELLULAR_RESPONSE_TO_TYPE_II_INTERFERON pathways altered by anti-PD1 in WT tumor cells (left) and the expression profile of the same genes in A159V tumor cells treated with IgG or anti-PD1 (right).

Fig. S11.

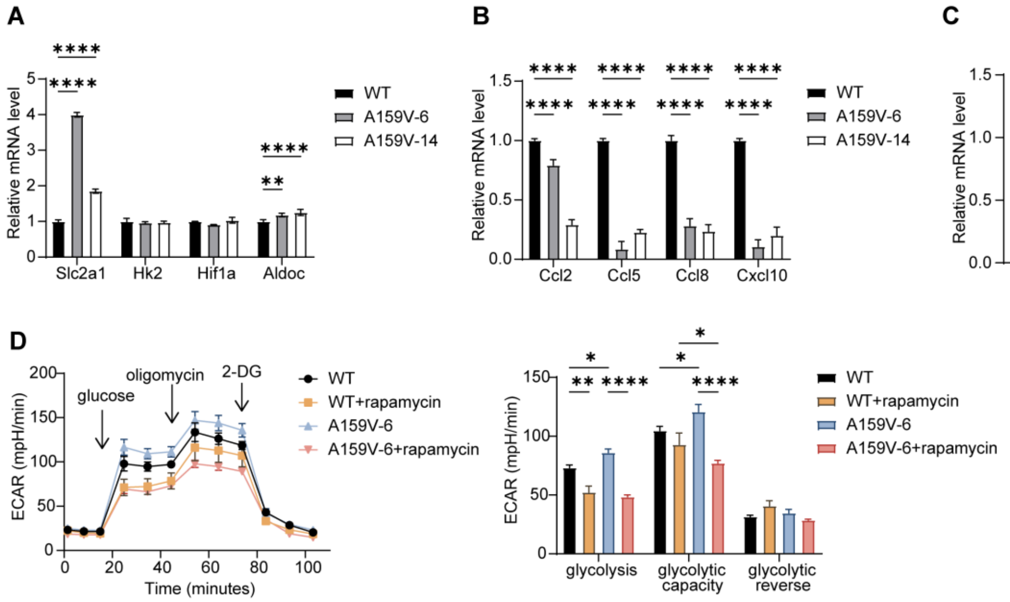

Fig. S11. Validation of RAC1^{A159V} downstream effects in different MC38 cell clones *in vitro*. (**A-C**) RT-qPCR analysis of selected gene expression in MC38 RAC1^{WT} and two more different RAC1^{A159V} cell clones (n=3). (A) Glycolysis-related genes, (B) chemokines, (C) Ifngr1. (**D**) Seahorse glycolysis stress test in MC38 RAC1^{WT} and RAC1^{A159V} clone 6 (n=2). Data represent mean ± s.d. Statistical significance determined by two-way ANOVA (**A, B, D**) and one-way ANOVA (**C**). *$P < 0.05$; **$P < 0.01$; ***$P < 0.001$; ****$P < 0.0001$.

Fig. S12.

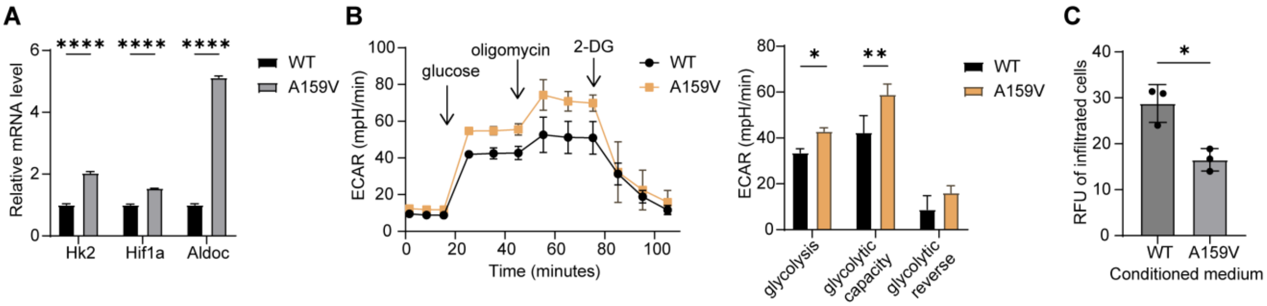

Fig. S12. Validation of RAC1^{A159V} downstream effects in B16OVA cells *in vitro.*

(**A**) RT-qPCR analysis of glycolysis-related gene expression in RAC1^{WT} and RAC1^{A159V} cells (n=3).

(**B**) Seahorse glycolysis stress test in RAC1^{WT} and RAC1^{A159V} cells (n=3). Data are representative of two independent experiments.

(**C**) Chemotaxis assay of CD8^{+} T cells towards conditioned medium derived from RAC1^{WT} and RAC1^{A159V} cells (n=3).

Data represent mean ± s.d. Statistical significance determined by two-way ANOVA (**A, B**) and unpaired t-test (**C**). *$P < 0.05$; **$P < 0.01$; ***$P < 0.001$; ****$P < 0.0001$.

Fig. S13.

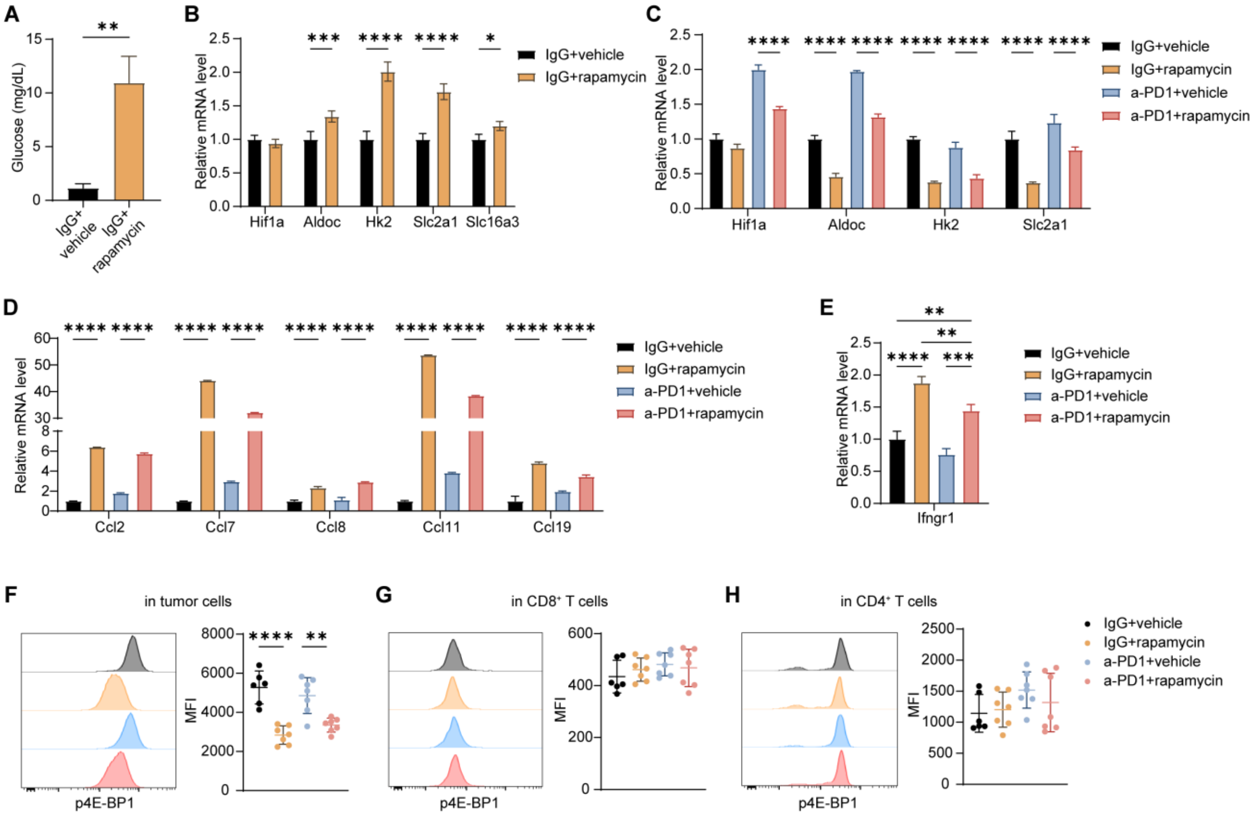

Fig. S13. Rapamycin treatment modulates RAC1^{A159V} tumor cell glycolysis, chemokine production and IFNGR1 expression *in vivo* without directly affecting T cells.

(**A**) Glucose concentration in the tumor interstitial fluid of RAC1^{A159V} tumors treated with vehicle or rapamycin (2 mg/kg) (n=3).

(**B**) RT-qPCR analysis of glycolysis-related gene expression in CD8^{+} T cells isolated from RAC1^{A159V} tumor tissues treated with vehicle or rapamycin (2 mg/kg) (n=3).

(**C-E**) RT-qPCR analysis of selected gene expression in tumor cells isolated from RAC1^{A159V} tumor tissues under indicated treatment (n=3). (C) Glycolysis-related genes, (D) chemokines, (E) Ifngr1.

(**F-H**) Flow cytometry analysis of phospho-4E-BP1 in tumor cells (F), CD8^{+} T cells (G) and CD4^{+} T cells (H) in RAC1^{A159V} tumors under indicated treatment (n=6-7 mice per group). Representative histogram (left) and MFI summary (right) are shown.

Data are representative of two independent experiments (**A**, **F-H**). Data represent mean ± s.d. Statistical significance determined by unpaired t-test (**A**), two-way ANOVA (**B-D**), and one-way ANOVA (**E-H**). *$P < 0.05$; **$P < 0.01$; ***$P < 0.001$; ****$P < 0.0001$.

Fig. S14.

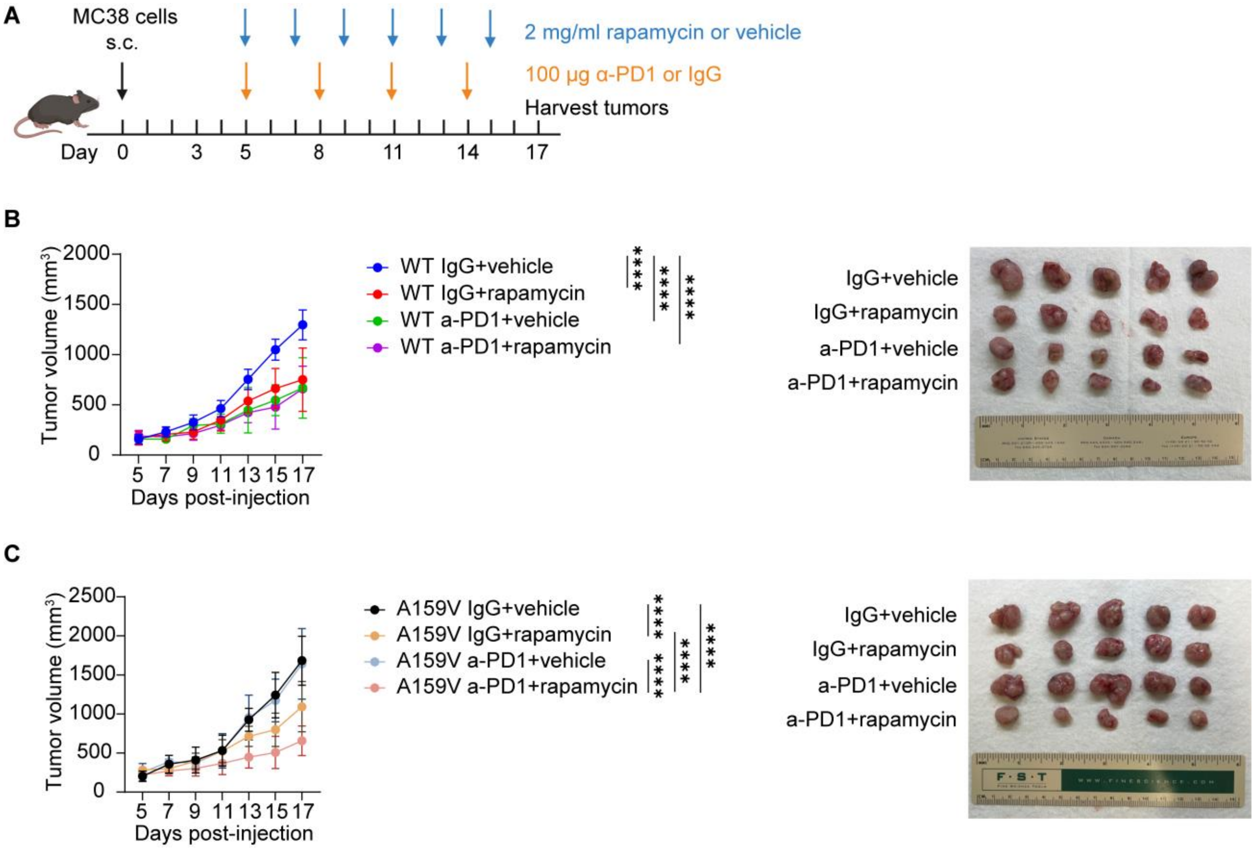

Fig. S14. Rapamycin does not augment the anti-tumor effects of anti-PD1 in RAC1^{WT} tumors
(**A**) Schematic illustration of tumor inoculation and drug administration. MC38 RAC1^{WT} and RAC1^{A159V} cells (2 million) were subcutaneously injected into C57BL/6 mice on day 0. Anti-PD1 or IgG isotype control (100 µg) was administrated intraperitoneally every three days starting from day 5. Rapamycin or vehicle (2 mg/kg) was administrated intraperitoneally every other day starting from day 5. Tumors were harvested on day 17.
(**B**) Tumor growth curve (left) and image (right) of RAC1^{WT} tumors (n=5 mice per group).
(**C**) Tumor growth curve (left) and image (right) of RAC1^{A159V} tumors (n=5 mice per group).
Data represent mean ± s.d. Statistical significance determined by two-way ANOVA. ****P < 0.0001.
